# Comparative efficacy of non-pharmacological therapies in adolescents with subthreshold depression: a systematic review and network meta-analysis

**DOI:** 10.3389/fpsyt.2026.1799128

**Published:** 2026-05-12

**Authors:** Qipei Ji, Zhenbo Wang, Xiaobo Wang, Ling Zhou, Zhenkui Lou, Meiling Su, Xin Gao, Shufang Jiang, Hongfei Qiu, Zhongchun Luo

**Affiliations:** Department of Rehabilitation Medicine, The People’s Hospital of Leshan, Leshan, China

**Keywords:** adolescents, network meta-analysis, non-pharmacological therapies, subthreshold depression, systematic review

## Abstract

**Background:**

Subthreshold depression (SD) is highly prevalent in adolescents and young adults aged 11–25 years, leading to impaired psychosocial functioning and a high risk of progression to major depressive disorder (MDD). Non-pharmacological therapies are the mainstay of intervention for adolescent SD, yet the comparative efficacy of different interventions remains unclear. This study aimed to compare the relative effectiveness of various non-pharmacological interventions for adolescent SD via network meta-analysis (NMA).

**Methods:**

We initially retrieved 5,297 records from six databases (PubMed, Embase, Web of Science, Cochrane, PsycINFO, CINAHL) for randomized controlled trials (RCTs) of non-pharmacological interventions for SD in individuals aged 11–25 years who did not meet the DSM-IV/ICD-10 diagnostic criteria for MDD or depressive episode. The control groups received waitlist control or usual care, and the primary outcome was depressive symptoms assessed by validated scales (BDI-II, CES-D, CDI, HAMD). Two reviewers independently conducted study selection, data extraction and risk of bias assessment (RoB 2.0). Heterogeneity was analyzed using the *I²* statistic; NMA was performed under a Bayesian framework with consistency assessment, and the CINeMA tool was used to grade the certainty of evidence. This study was registered in PROSPERO (CRD42023456264) and reported in accordance with the PRISMA 2020 statement.

**Results:**

A total of 30 RCTs involving 3,471 participants were included. Traditional meta-analysis showed that non-pharmacological interventions significantly improved depressive symptoms in adolescents with SD (pooled SMD = −0.93, 95% CI: −1.20 to −0.66, *P* < 0.0001), with high heterogeneity across studies (*I²* = 92%) driven by intervention type, delivery mode, intervention duration and participant age. NMA results indicated that behavioral activation (BA) was the most effective intervention (SMD = −3.45, 95% CI: −4.89 to −2.02), followed by physical exercise intervention (PEI) (SMD = −1.34, 95% CI: −2.68 to −0.03) and light therapy (LT) (SMD = −1.24, 95% CI: −2.17 to −0.31). No significant overall inconsistency was detected in the study (χ²=6.72, df=9, *P* = 0.651).

**Conclusions:**

Non-pharmacological interventions are effective for adolescents with SD, and BA is recommended as the first-line clinical option. Clinical selection of interventions should take into account individual patient characteristics and intervention features. This study has several limitations, including high heterogeneity across studies, single-study evidence for some interventions (e.g., LT, PEI), a broad age range of participants and the lack of long-term follow-up data. Further large-scale, multi-center RCTs are needed to validate these findings.

## 1 Introduction

The most widely used definition of subthreshold depression (SD) is a clinically relevant level of depressive symptoms that does not meet the diagnostic criteria for major depressive disorder (MDD) in DSM-IV (or depressive episode in ICD-10). Meanwhile, SD can be defined by dimension and category: dimension, i.e., by a cutoff score on a widely validated depression scale in the absence of meeting diagnostic criteria for MDD; and category, i.e., fewer than 5 DSM-IV symptoms (1, 2).

SD represents a major public health burden among adolescents, with epidemiologic data highlighting its high prevalence and severe clinical impacts. Epidemiologic surveys in the US show that 20% of the general population has SD symptoms (3)., while among adolescents, the prevalence reaches up to 29.2% (4)—a rate significantly higher than in other age groups, with prevalence peaking during this critical developmental stage (4, 5). Beyond high prevalence, adolescents with SD face substantial adverse outcomes: they exhibit poorer quality of life, varying degrees of psychosocial and socio-emotional dysfunction, and significantly higher suicidal ideation compared to non-depressed peers (6–8). One study confirmed that quality of life is lower in individuals with SD than in non-depressed populations, and lowest in those with MDD (9). More critically, SD is a strong precursor to full-blown MDD: a longitudinal study found that over half of adolescents with SD before age 30 will develop MDD by an average age of 22.2 years (10), and numerous studies have verified that people with SD have a significantly higher incidence of MDD than healthy individuals without SD (11). These findings underscore the urgent need for effective interventions to mitigate the short- and long-term burden of SD in adolescents.

Therefore, identifying optimal treatments for adolescent SD is clinically crucial (12). Various non-pharmacological treatment strategies have been explored, including cognitive behavioral therapy, behavioral activation therapy, web-based coaching interventions, physical exercise, positive thinking therapy, counseling therapy, and interpersonal therapy (13–15). Existing evidence confirms that these interventions improve depressive symptoms to varying degrees and reduce the risk of MDD progression (13). For example, a meta-analysis of 700 patients concluded that psychotherapy is more beneficial than conventional treatment for SD (16), while a meta-analysis of internet-based interventions demonstrated their efficacy in alleviating depressive symptoms relative to conventional therapy (17). However, pharmacological treatments for SD have been seriously questioned, with a systematic review concluding no significant advantage of antidepressants over placebo (13).

Despite the availability of multiple non-pharmacological options, critical gaps remain in the evidence base. Previous pairwise meta-analyses have only compared individual interventions against controls or limited head-to-head comparisons, failing to comprehensively rank the relative efficacy of diverse therapies (16, 17). Notably, no prior network meta-analysis (NMA) has systematically synthesized data to compare the comparative effectiveness of various non-pharmacological interventions specifically in the adolescent SD population. Compared to pairwise meta-analysis, NMA offers a unique advantage by integrating direct and indirect evidence from multiple trials, enabling simultaneous comparison of multiple interventions and generating relative efficacy rankings—information that is invaluable for clinical decision-making. Given the lack of systematic comparative evidence for non-pharmacological therapies in adolescent SD, this study conducted a systematic review and NMA to comprehensively compare the efficacy of various non-pharmacological interventions, aiming to fill this evidence gap and provide actionable guidance for clinical practice.

## 2 Materials and methods

This SR and Meta-analysis protocol is registered with the International Prospective Register of Systematic Reviews (PROSPERO) (Registration CRD42023456264). We performed this SR and Meta-analysis according to A Measurement Tool to Assess Systematic Reviews 2 (AMSTAR 2) (18) and reported according to the Preferred Reporting Items for Systematic Reviews and Meta-Analysis (PRISMA) 2020 statement (**Supplementary Appendix 1**) (19).

### 2.1 Search strategy

We searched six electronic databases (PubMed, Embase, Web of Science, Cochrane Library, PsycINFO, CINAHL) from January 1, 2005, to December 31, 2025. The search combined three core concepts with Boolean logic: subthreshold depression and synonyms (OR-connected), adolescents/young adults (OR-connected), and RCTs (OR-connected), with concepts linked by AND. Manual reference list searching and expert consultation were also conducted. Full search strategies are provided in **Supplementary Appendix 2**.

### 2.2 Inclusion and exclusion criteria

#### 2.2.1 Inclusion criteria

Studies were selected if they met the following inclusion criteria:

1. Participants: Adolescents and young adults aged 11–25 years (20) with clinically relevant depressive symptoms that do not meet the full diagnostic criteria for major depressive disorder (MDD) according to DSM-IV or ICD-10 diagnostic criteria (1, 2).
2. Interventions: Non-pharmacological interventions were administered to the experimental group, including cognitive–behavioral intervention (internet-based cognitive behavioral therapy, group cognitive behavioral therapy, cognitive bibliotherapy), behavioral activation, attentional bias modification, interpersonal psychotherapy-adolescent skills training, positive intervention, school counseling, health education, mindfulness-based intervention, smartphone application, school-based group coping skills, physical exercise intervention, bright light therapy and dim light therapy.
3. Comparators: The control group received either waitlist control or usual care.
4. Outcomes: Depressive symptoms were measured by validated scales including the Beck Depression Inventory-II (BDI-II), the Center for Epidemiological Studies Depression Scale (CES-D), the Hamilton Depression Rating Scale (HAM-D), and the Children’s Depression Inventory (CDI). At least one of the above scales was used for outcome assessment.
5. Study design: Randomized controlled trials (RCTs).

#### 2.2.2 Exclusion criteria

Studies were excluded if they met any of the following criteria: (1) Conference papers, abstracts, animal experiments, duplicate publications, systematic reviews, and meta-analyses; (2) Unavailable objective data for effect size calculation, data loss, or inaccessible full text even after contacting the corresponding author; (3) Non-English literature; (4) Studies involving participants aged under 11 years or over 25 years.

### 2.3 Study selection

Endnote 20 was utilized to manage the searched results of the records. Following removing duplicate studies, two reviewers independently screened titles and abstracts for potentially eligible studies based on the inclusion criteria. They then read the full text of potentially eligible studies to determine the final included literature. A third reviewer was consulted for any disagreements.

### 2.4 Outcome measures

The primary outcome was depressive symptoms, evaluated using validated standardized scales: the Beck Depression Inventory-II (BDI-II), Center for Epidemiologic Studies Depression Scale (CES-D), Children’s Depression Inventory (CDI), and Hamilton Depression Rating Scale (HAMD). Higher scores on these scales reflect more severe depressive symptoms.

The effect measure for the primary outcome was the standardized mean difference (SMD) with 95% credible intervals (CrI).

### 2.5 Data collection

Two researchers independently screened titles and abstracts, and all potentially eligible studies were reviewed in full text according to the inclusion criteria. Data were extracted using standardized tables, including information on population characteristics, interventions, comparisons, outcomes, and other relevant details (e.g., study design, publication year, measurement tools, statistical methods, etc.).

### 2.6 Risk of bias and quality of evidence grading

Two researchers (Q-p J and Z-b W) independently assessed the risk of bias using the Cochrane Risk of Bias Assessment Tool (RoB 2.0), covering six domains: selection bias (random sequence generation, allocation concealment), performance bias (blinding of participants and staff), detection bias (blinding of outcome assessment), attrition bias (incomplete outcome data), reporting bias (selective reporting), and other biases. Each domain was rated as low, high, or unclear risk.

Evidence quality was graded with the CINeMA tool, which comprehensively evaluates network meta-analysis evidence from six aspects (within-study bias, across-studies bias, indirectness, imprecision, heterogeneity, incoherence) and categorizes each as not serious, serious, or very serious. The final evidence certainty was rated as High, Moderate, Low, or Very Low.

All assessments were cross-checked by the two researchers, with discrepancies resolved through consultation with a third independent reviewer.

### 2.7 Statistical analysis

Statistical analyses were conducted using STATA 14.0. The primary outcome (depressive symptoms) was a continuous variable, and network meta-analysis (NMA) was performed within a Bayesian Markov chain Monte Carlo (MCMC) framework to estimate intervention effects and plot network relationships.

Consistency was evaluated using an inconsistency model; a consistency model was applied if the overall inconsistency test yielded *P* > 0.05 (indicating no significant overall inconsistency). Local inconsistency was assessed via the node-splitting method, with *P* > 0.05 indicating good agreement between direct and indirect comparisons. For closed loops in the network, loop inconsistency (LF) factors were used to identify inconsistency; consistency between direct and indirect evidence was confirmed if the lower limit of the 95% confidence interval (CI) of the inconsistency factor (IF) was zero or near zero.

Intervention rankings were determined using the surface under the cumulative ranking curve (SUCRA) (range: 0–1). Additionally, publication bias was assessed using comparison-adjusted funnel plots in STATA 14.0 to verify the robustness of the NMA results.

## 3 Results

### 3.1 Research identification and selection

**Figure 1:**
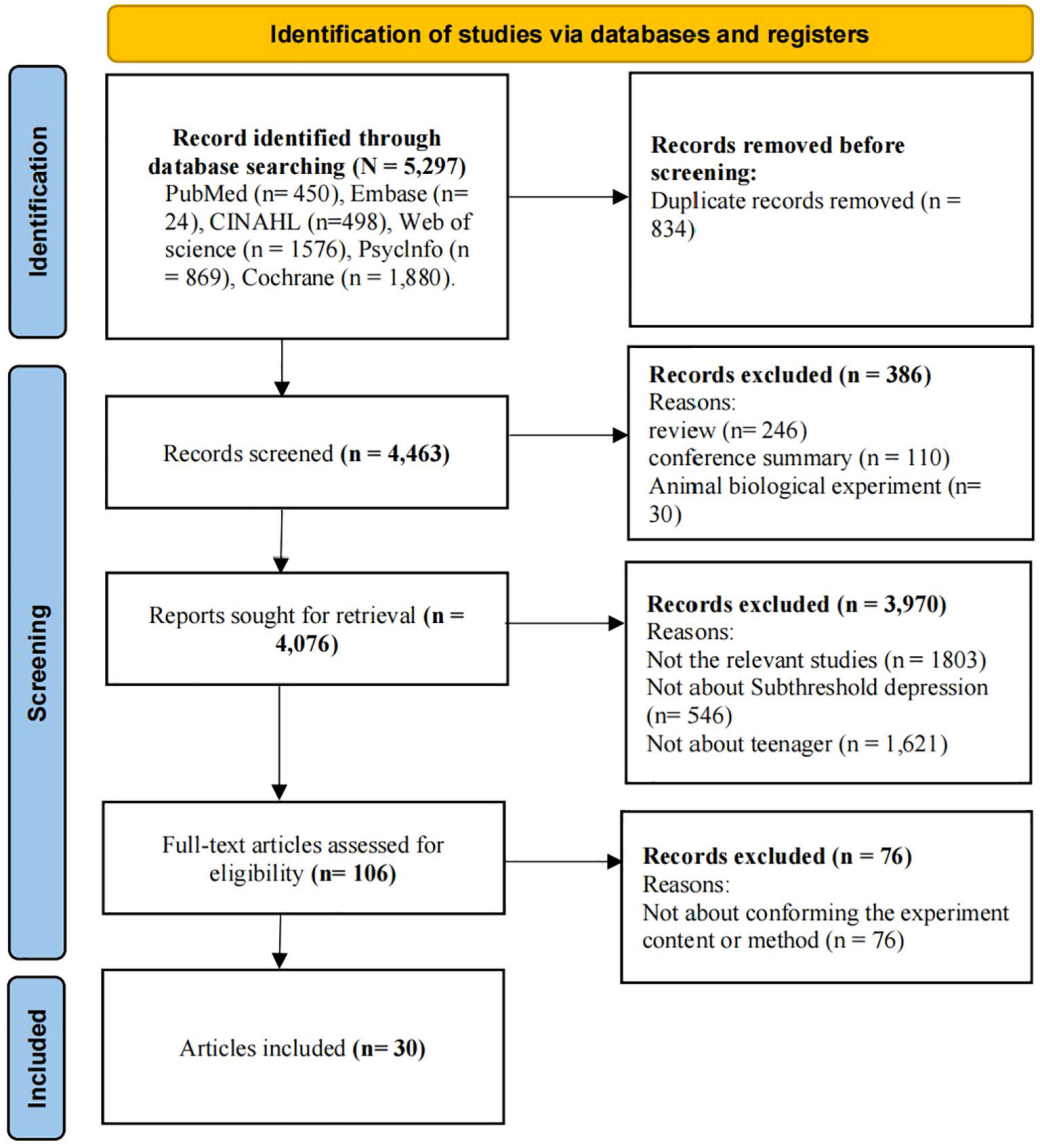
Flow diagram of study selection according to the PRISMA 2020 statement.

Approximately 5,297 studies from six databases were initially retrieved. After duplicate removal, screening of publication types, title/abstract review, and full-text evaluation, 30 independent RCTs were ultimately included for the network meta-analysis (**Figure 1**) (21–50). Among these, 27 RCTs with a control arm matching the definition of usual care/waitlist control were included in the traditional meta-analysis, with some trials contributing multiple comparison arms, resulting in a total of 31 effect sizes for the pairwise meta-analysis. The remaining 3 RCTs (25, 30, 42) were excluded from the traditional meta-analysis because their control groups did not meet the criterion of conventional care (e.g., using active control or non-standard care designs).

### 3.2 Characteristics of the included studies

**Table 1** presents the detailed methodological and demographic characteristics of the 30 independent RCTs included in this systematic review and network meta-analysis. Three of these trials employed multiple comparison arms, resulting in a total of 31 effect sizes for the conventional pairwise meta-analysis. The studies were conducted across 18 countries, with the largest contributions from the United States, Australia, China, Japan, the Netherlands, and South Korea.

**Table 1:** Characteristics of included studies.

Study ID	Country/ region	Setting	Sample size (T/C)	Mean age (T/C, years)	Sex distribution (F/M, %)	Baseline depression score (T/C, mean ± SD)	Course of treatment	Follow-up duration	Dropout/attrition rate (T/C, %)	Assessment scales
22	USA	School (in-school)	48/46	13.7 ± 1.2 / 13.9 ± 1.3	64.6/35.4 / 62.2/37.8	CES-D: 18.2 ± 8.5 / 17.8 ± 8.3	4 weeks	18 months	10.4 (5/48) / 13.0 (6/46)	BDI-II
23	Croatia	Community mental health center	27/25	18.7 ± 2.3 / 18.9 ± 2.2	59.3/40.7 / 56.0/44.0	BDI-II: 13.2 ± 6.1 / 13.5 ± 6.3	6 months	6 months	11.1 (3/27) / 12.0 (3/25)	CDI
24	Germany	School (after-school)	34/32	15.8 ± 1.4 / 15.6 ± 1.5	61.8/38.2 / 59.4/40.6	CES-D: 16.5 ± 7.8 / 16.1 ± 7.6	4 weeks	6 months	11.8 (4/34) / 12.5 (4/32)	BDI-II
25	USA	School (in-school)	41/39	14.2 ± 1.8 / 14.5 ± 1.9	63.4/36.6 / 61.5/38.5	CES-D: 24.6 ± 6.9 / 23.8 ± 7.2	6 months	6 months	7.3 (3/41) / 10.3 (4/39)	BDI-II
21	United Kingdom	Hospital/clinic (pediatric obesity clinic)	28/23/30 (3 arms)	13.1 ± 1.5 / 13.3 ± 1.4 / 13.0 ± 1.6	55.6/44.4 / 56.5/43.5 / 53.3/46.7	CDI: 10.1 ± 8.18 / 9.1 ± 6.37 / 8.9 ± 6.21	8 weeks	28 months	7.1 (2/28) / 8.7 (2/23) / 16.7 (5/30)	BDI-II
26	USA	Clinical setting (university outpatient)	36/34	17.2 ± 2.1 / 17.5 ± 2.0	63.9/36.1 / 61.8/38.2	BDI-II: 16.4 ± 6.8 / 16.1 ± 6.6	8 weeks	9 months	8.3 (3/36) / 11.8 (4/34)	CES-D
27	USA	Hospital gastroenterology clinic	22/19	14.95 ± 2.33 / 15.02 ± 1.83	54.5/45.5 / 47.5/52.5	CDI: 25.7 ± 10.8 / 21.8 ± 8.1 (CDI-CP	12 weeks	Post-treatment only	13.6 (3/22) / 0 (0/19)	BDI-II
28	USA	School (in-school)	55/53	15.6 ± 1.5 / 15.4 ± 1.6	67.3/32.7 / 65.1/34.9	CES-D: 17.6 ± 8.2 / 17.3 ± 8.4	6 months	6 months	7.3 (4/55) / 9.4 (5/53)	CES-D
29	USA	School (in-school/after-school)	63/61	14.3 ± 1.4 / 14.5 ± 1.5	65.1/34.9 / 63.6/36.4	CES-D: 16.8 ± 8.1 / 16.5 ± 8.3	8 weeks	24 months	11.1 (7/63) / 13.1 (8/61)	CDI
30	USA	School (after-school)	58/55	13.8 ± 1.3 / 13.6 ± 1.4	65.5/34.5 / 63.6/36.4	CES-D: 16.2 ± 8.3 / 15.7 ± 8.5	6 months	12 months	10.3 (6/58) / 12.7 (7/55)	BDI-II
31	Romania	School (after-school)	29/27	15.4 ± 1.3 / 15.2 ± 1.4	62.1/37.9 / 60.7/39.3	CES-D: 15.9 ± 7.4 / 15.6 ± 7.2	1 month	6 months	13.8 (4/29) / 14.8 (4/27)	BDI-II
33	Netherlands	Online (community-supported)	33/31	19.8 ± 2.2 / 20.1 ± 2.3	72.7/27.3 / 70.9/29.1	–	4 weeks	8 months	15.2 (5/33) / 16.1 (5/31)	CES-D
32	India	University campus	35/33	22.3 ± 2.5 / 22.1 ± 2.4	65.7/34.3 / 63.6/36.4	BDI-II: 15.7 ± 6.5 / 15.3 ± 6.7; CES-D: 24.8 ± 7.0 / 24.3 ± 6.8	8 weeks	3 months	11.4 (4/35) / 12.1 (4/33)	CDI
35	China	University psychology lab	27/27/23 (3 arms)	19.44 ± 1.58 / 19.52 ± 0.89 / 19.57 ± 0.73	74.1/25.9 / 63.0/37.0 / 78.3/21.7	BDI-II: 17.33 ± 3.81 / 18.04 ± 4.11 / 18.13 ± 5.18	4 weeks	7 months	14.8 (4/27) / 22.2 (6/27) / 21.7 (5/23)	CES-D
34	USA	University medical centers/community mental health center	159/157	15.0 ± 1.1 / 15.2 ± 1.2	52.2/47.8 / 53.5/46.5	CES-D: 22.3 ± 4.6 / 21.9 ± 4.8	3 months	75 months	12.6 (20/159) / 14.0 (22/157)	CES-D
36	Japan	University health service center	62/56	18.19 ± 0.41 / 18.21 ± 0.43	37.1/62.9 / 39.3/60.7	BDI-II: 12.83 ± 6.71 / 13.27 ± 6.02	5 weeks	6 months	8.1 (5/62) / 7.1 (4/56)	CES-D
37	USA	School (in-school/after-school)	95/91	13.56 ± 1.28 / 13.42 ± 1.18	67.4/32.6 / 65.9/34.1	CES-D: 15.51 ± 8.52 / 15.07 ± 8.65	6 months	6 months	5.3 (5/95) / 6.6 (6/91)	CES-D
38	Netherlands	Online (home-based) + School (assessment)	38/32/38 (3 arms)	14.73 ± 1.57 / 14.31 ± 1.51 / 14.29 ± 1.50	63.2/36.8 / 65.6/34.4 / 71.1/28.9	CDI: 14.32 ± 7.39 / 11.50 ± 6.40 / 12.71 ± 7.29	8 weeks	6 months	15.8 (6/38) / 18.8 (6/32) / 13.2 (5/38)	CES-D
39	Australia	School (in-school)	44/42	14.9 ± 1.6 / 15.1 ± 1.5	65.9/34.1 / 64.3/35.7	BDI-II: 13.7 ± 5.9 / 13.4 ± 5.7	4 weeks	12 months	9.1 (4/44) / 9.5 (4/42)	HAMD
41	Japan	University health service center	62/56	18.23 ± 0.42 / 18.20 ± 0.40	38.7/61.3 / 37.5/62.5	BDI-II: 12.76 ± 6.66 / 13.30 ± 5.95	5 weeks	1 year	16.1 (10/62) / 10.7 (6/56)	BDI-II
40	India	University campus	40/38	21.9 ± 2.3 / 22.2 ± 2.5	67.5/32.5 / 65.8/34.2	BDI-II: 14.9 ± 6.3 / 15.2 ± 6.1; CES-D: 24.5 ± 6.9 / 24.1 ± 6.7	8 weeks	6 months	10.0 (4/40) / 10.5 (4/38)	BDI-II
45	Brazil	University health service center	30/28	21.4 ± 2.8 / 21.7 ± 2.6	63.3/36.7 / 60.7/39.3	BDI-II: 15.3 ± 6.8 / 14.9 ± 6.6; CES-D: 25.1 ± 7.2 / 24.6 ± 7.0	8 weeks	6 months	13.3 (4/30) / 14.3 (4/28)	BDI-II
42	USA	School (in-school/after-school)	112/108	14.1 ± 1.5 / 14.3 ± 1.6	64.3/35.7 / 62.9/37.1	CES-D: 15.9 ± 8.7 / 15.3 ± 8.9	6 months	24 months	12.5 (14/112) / 14.8 (16/108)	CES-D
43	China	University counseling center	45/43	20.8 ± 2.1 / 21.1 ± 2.3	71.1/28.9 / 69.8/30.2	BDI-II: 14.8 ± 6.4 / 14.5 ± 6.2; CES-D: 26.5 ± 7.3 / 25.9 ± 7.5	8 weeks	6 months	8.9 (4/45) / 9.3 (4/43)	CES-D
46	Iran	University counseling center	28/26	20.3 ± 2.2 / 20.6 ± 2.3	67.9/32.1 / 65.4/34.6	BDI-II: 14.5 ± 6.4 / 14.2 ± 6.5	8 weeks	4 months	10.7 (3/28) / 11.5 (3/26)	CES-D
44	China	University dormitories (self-administered light therapy)	50/50/42 (3 arms)	21.18 ± 2.31 / 21.49 ± 2.35 / 21.38 ± 2.22	68.6/31.4 / 74.5/25.5 / 64.3/35.7	BDI-II: 23.22 ± 6.49 / 21.75 ± 7.72 / 21.81 ± 6.21; HAMD: 14.75 ± 3.35 / 13.75 ± 3.55 / 14.45 ± 3.76; CES-D: 24.9 ± 6.8 / 23.8 ± 7.1 / 23.6 ± 6.9	8 weeks	Post-treatment only	2 (1/51) / 2 (1/51) / 0 (0/42)	BDI-II
47	South Korea	University mental health clinic	39/37	21.5 ± 2.4 / 21.3 ± 2.5	69.2/30.8 / 67.6/32.4	BDI-II: 14.8 ± 6.3 / 14.5 ± 6.2; CES-D: 25.3 ± 7.1 / 24.8 ± 7.3	8 weeks	6 months	10.3 (4/39) / 11.0 (4/37)	CDI
48	Japan	Community mental health center	32/30	19.5 ± 2.4 / 19.8 ± 2.2	56.2/43.8 / 53.3/46.7	BDI-II: 13.6 ± 6.2 / 14.1 ± 6.4	5 weeks	9 months	12.5 (4/32) / 13.3 (4/30)	CDI
49	Finland	School (in-school)	38/36	15.3 ± 1.2 / 15.1 ± 1.3	60.5/39.5 / 58.3/41.7	BDI-II: 14.2 ± 5.8 / 13.8 ± 5.6	9 weeks	12 months	7.9 (3/38) / 8.3 (3/36)	BDI-II
50	China	University counseling center	43/41	20.9 ± 2.2 / 21.2 ± 2.4	70.2/29.8 / 68.3/31.7	BDI-II: 14.6 ± 6.5 / 14.3 ± 6.3; CES-D: 25.1 ± 7.2 / 24.7 ± 7.4	2 weeks	6 months	9.3 (4/43) / 9.8 (4/41)	BDI-II

BDI-II, Beck Depression Inventory-II; CES-D, Center for Epidemiologic Studies Depression Scale; CDI, Children's Depression Inventory; HAMD, Hamilton Depression Rating Scale; T, Non-pharmacological intervention group; C, Control group; SD, Standard deviation. Multi-arm sample size follows "Intervention/Placebo/Control"; baseline scores include only the above scales (marked "-" if unavailable); dropout rate, dropouts/total participants; setting indicates core implementation environments.

The combined sample comprised 3,471 participants aged 11 to 25 years—encompassing adolescents and young adults undergoing critical transitions in psychological and social development. All participants met established criteria for subthreshold depression, defined as clinically significant depressive symptoms that do not satisfy the full diagnostic criteria for major depressive disorder according to the Diagnostic and Statistical Manual of Mental Disorders, Fifth Edition (DSM-5). Participants were randomly assigned to either non-pharmacological intervention groups—including cognitive behavioral therapy (CBT), attention bias modification (ABM), bright light therapy, and school-based group interventions—or control conditions (waitlist and treatment-as-usual).

Key trial characteristics—including mean age, sex distribution, intervention setting (school, clinic, community, online, or home-based), baseline depression severity (assessed using standardized instruments: BDI-II, CES-D, CDI, or HAMD), intervention duration, follow-up period, and participant retention/dropout rates—are comprehensively reported in **Table 1**. **Supplementary Table 2** summarizes comprehensive study-level information, including diagnostic definitions of subthreshold depression and detailed intervention characteristics (type, delivery mode, duration, core components, provider background, and adherence monitoring) for all included trials.

### 3.3 Traditional meta-analysis results

#### 3.3.1 Overall intervention effect

**Figure 2:**
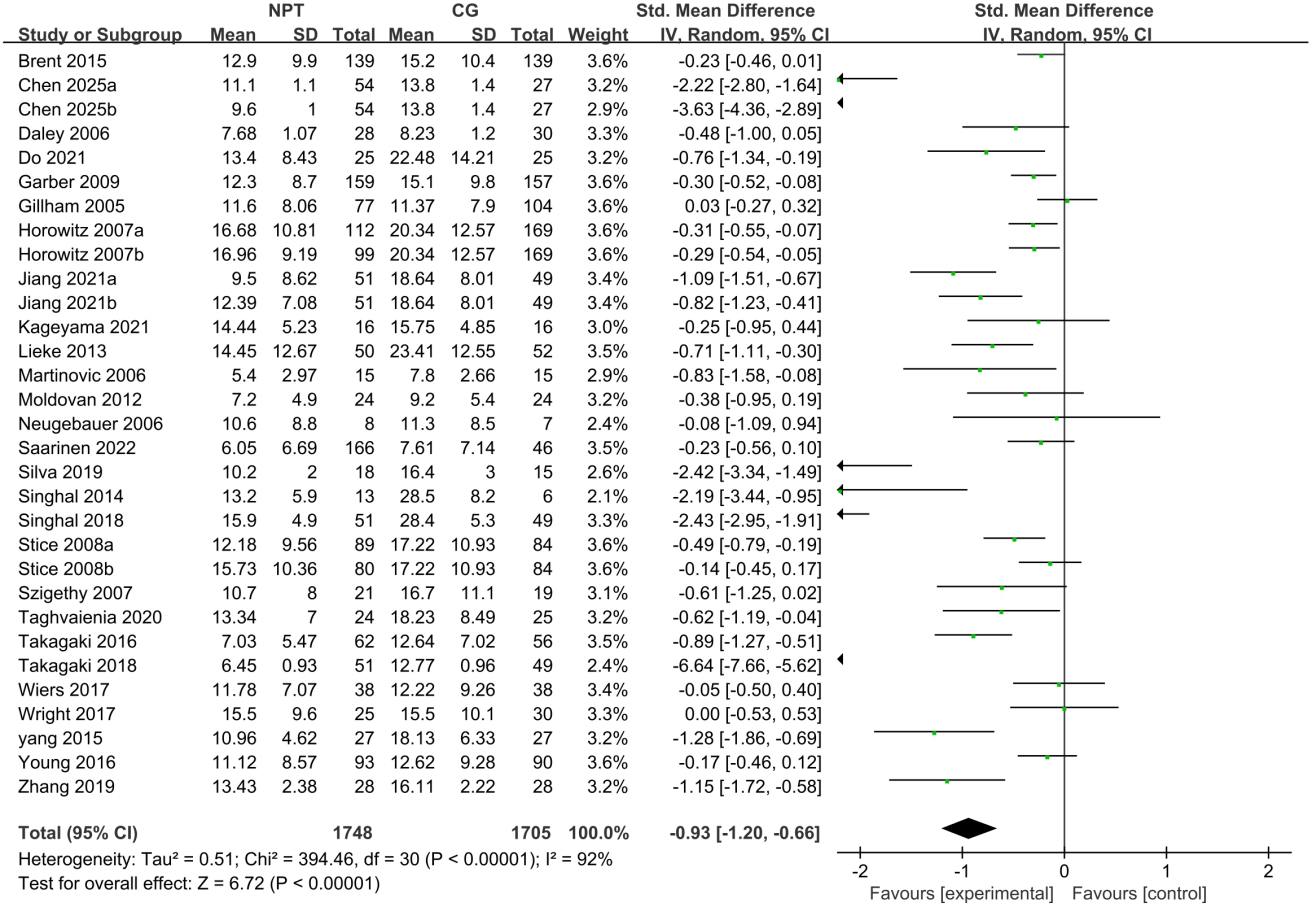
Forest plot of meta-analysis for non-pharmacological interventions on adolescent subthreshold depression.

A total of 27 studies meeting the inclusion criteria for traditional pairwise meta-analysis were screened out from the 30 included independent RCTs, with the remaining 3 studies excluded due to their control groups not adopting the usual care/waitlist control design (25, 30, 42) (**Figure 2**). All participants enrolled in these 27 studies were adolescents (including early young adults) aged 11 to 25 years with subthreshold depression. The random-effects model was used to pool the effect sizes, and the results showed that non-pharmacological interventions significantly improved depressive symptoms in this population, with a pooled standardized mean difference (SMD) of −0.93 (95% CI: −1.20 to −0.66, Z = 6.72, *P* < 0.0001). According to Cohen’s criteria for effect size classification, this indicated a moderate to large effect of non-pharmacological interventions on alleviating depressive symptoms in adolescents with subthreshold depression aged 11 to 25 years. Heterogeneity test revealed high heterogeneity across the included studies (*I²* = 92%, χ² = 394.46, df = 30, *P* < 0.0001). Leave-one-out sensitivity analysis demonstrated that the overall effect remained statistically significant after excluding any single study (all P < 0.0001), confirming the good robustness of the conclusion. Notably, the exclusion of Takagaki 2018 resulted in a pooled SMD of −0.77 (95% CI: −0.99 to −0.54) and a reduction in heterogeneity to 89%, suggesting this study may be a key source of the high heterogeneity. The aforementioned heterogeneity may also be attributed to multiple factors including intervention types, delivery settings, follow-up durations and age subgroups of the study population, which will be further explored in subsequent subgroup analyses.

#### 3.3.2 Subgroup analysis

##### 3.3.2.1 Subgroup analysis by intervention type

**Figure 3:**
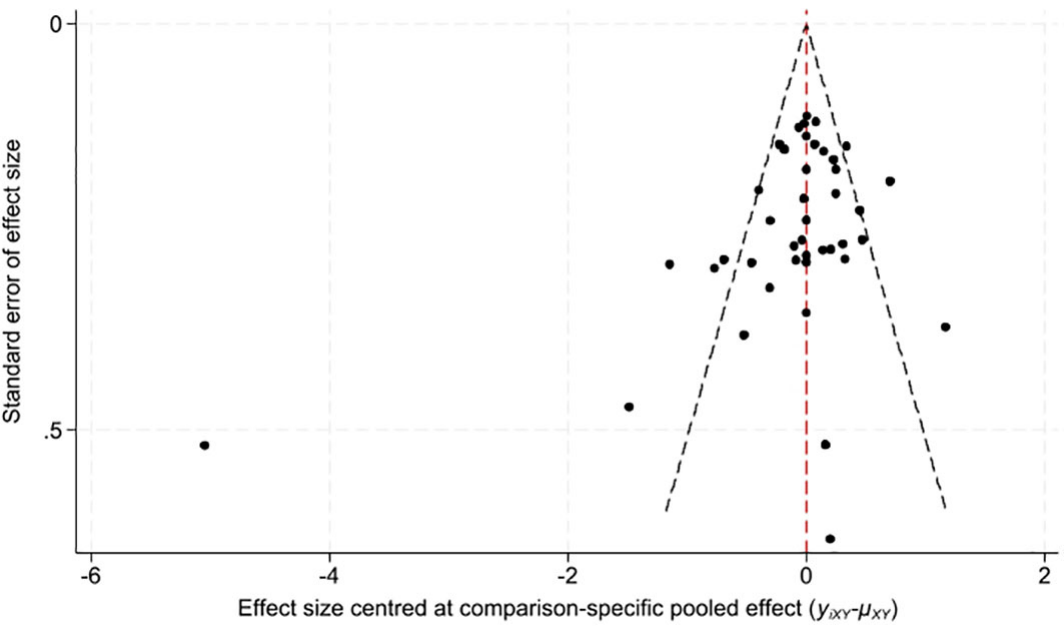
Forest plot of subgroup analysis stratified by intervention type.

Subgroup analyses were conducted using a random-effects model (**Figure 3**). Cognitive Behavioral Therapy (CBT, 14 studies) yielded a pooled SMD of −1.04 (95% CI: −1.43 to −0.66, Z = 5.32, *P* < 0.0001, *I²* = 95%); Mindfulness-Based Intervention (MBI, 4 studies) showed a pooled SMD of −1.47 (95% CI: −2.43 to −0.51, Z = 3.00, *P* = 0.003, *I²* = 91%); Physical Exercise Intervention (1 study) also showed no significance (SMD = −0.48, 95% CI: −1.00 to 0.05, *P* = 0.07); Other Interventions (9 studies) yielded a pooled SMD of −0.44 (95% CI: −0.72 to −0.15, Z = 3.01, *P* = 0.003, *I²* = 63%). The results of the between-group difference test (χ² = 9.40, df = 3, *P* = 0.02, *I²* = 68.1%) demonstrated that the effects of different intervention types were significantly distinct, and the intervention type was a crucial source of overall heterogeneity.

##### 3.3.2.2 Subgroup analysis by delivery mode

**Figure 4:**
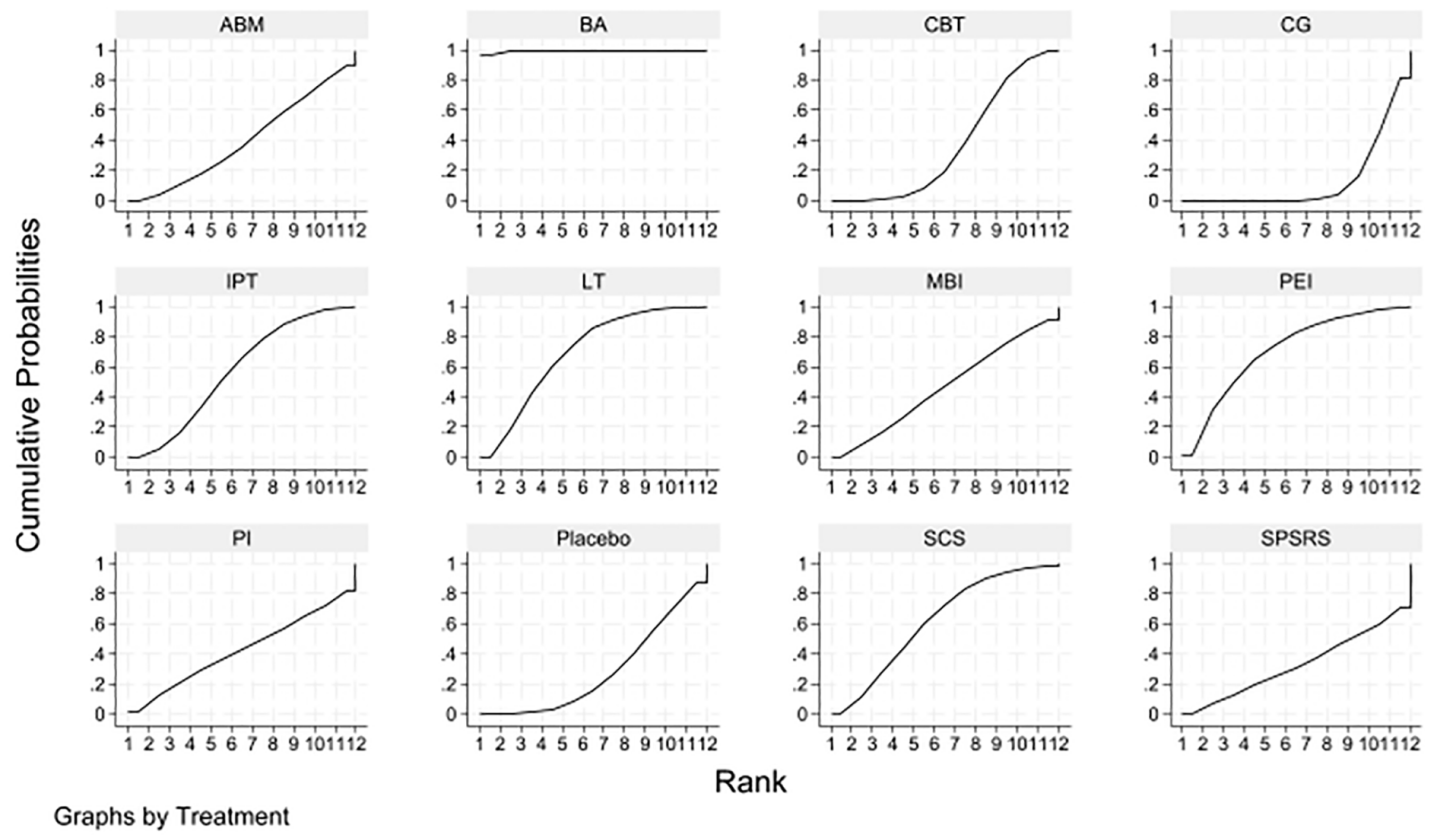
Forest plot of subgroup analysis stratified by delivery mode.

Subgroup analyses were conducted using a random-effects model (**Figure 4**). School-based intervention (8 studies) yielded a pooled SMD of −0.22 (95% CI: −0.33 to −0.11, Z = 3.89, *P* = 0.0001, *I²* = 0%); clinical-based intervention (12 studies) showed a pooled SMD of −1.45 (95% CI: −2.00 to −0.90, Z = 5.19, *P* < 0.0001, *I²* = 95%); home online intervention (2 studies) yielded a pooled SMD of −0.68 (95% CI: −1.00 to −0.35, Z = 4.04, *P* < 0.0001, *I²* = 0%); combined mode intervention (4 studies) yielded a pooled SMD of −1.24 (95% CI: −2.48 to −0.00, Z = 1.96, *P* = 0.05, *I²* = 94%). The results of the between-group difference test (χ² = 26.23, df = 3, *P* < 0.0001, *I²* = 88.6%) demonstrated that the effects of different delivery modes were significantly distinct, and delivery mode was a crucial source of overall heterogeneity.

##### 3.3.2.3 Subgroup analysis by intervention duration

Intervention duration was categorized into short-term (≤4 weeks), medium-term (5–8 weeks) and long-term (>8 weeks) based on mainstream research conventions in adolescent non-pharmacological depression intervention (45, 51) and the natural clustering characteristics of intervention durations in the included 30 RCTs. This classification is consistent with clinical practice guidelines for adolescent mental health, corresponding to the acute, standard and consolidation phases of non-pharmacological depression intervention with clear clinical significance.

**Figure 5:**
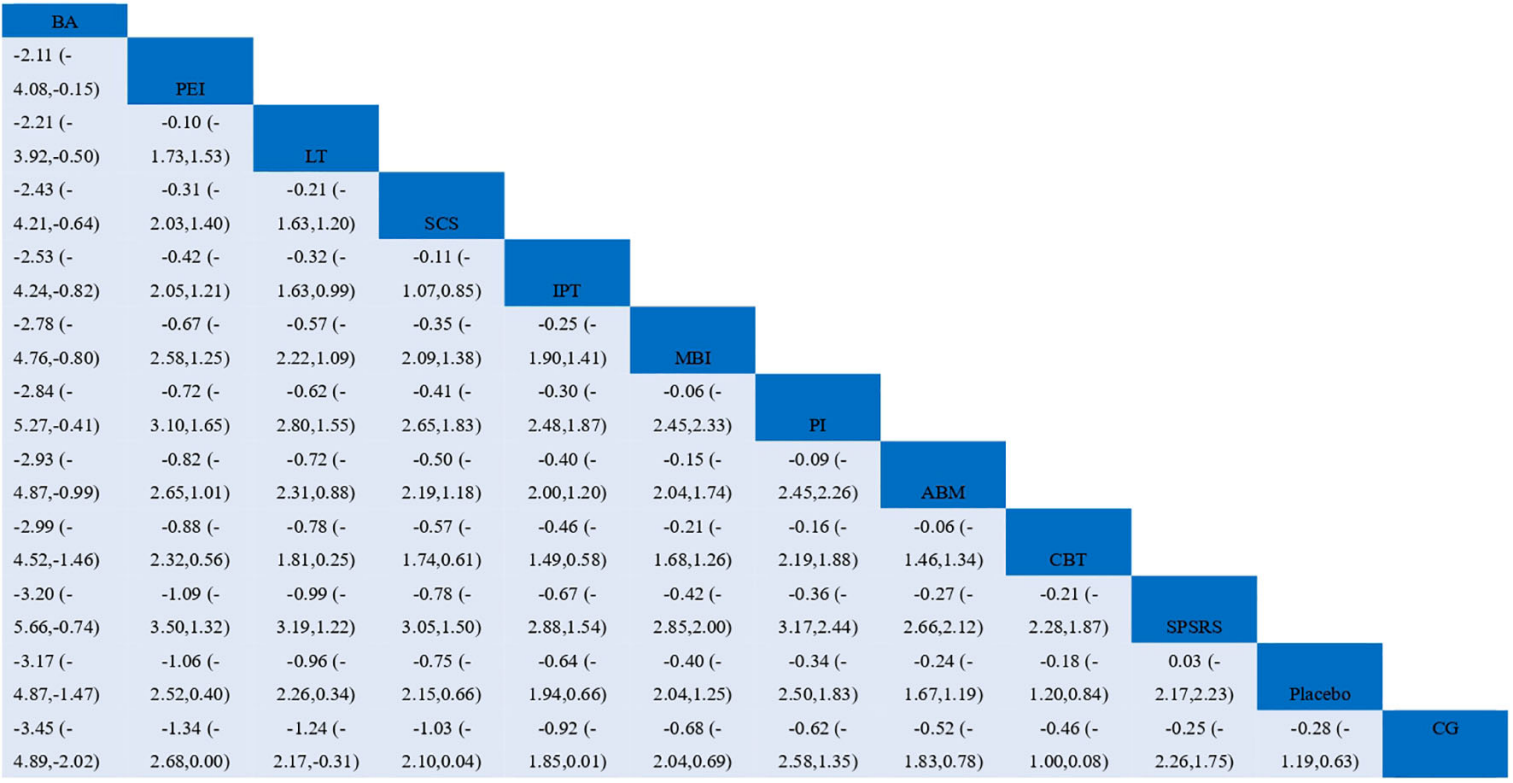
Forest plot of subgroup analysis stratified by intervention duration.

Subgroup analyses were conducted using a random-effects model (**Figure 5**). Short-term intervention (≤4 weeks, 8 studies) yielded a pooled SMD of −0.86 (95% CI: −1.36 to −0.36, Z = 3.37, *P* = 0.0008, *I²* = 93%); medium-term intervention (5–8 weeks, 9 studies) showed a pooled SMD of −1.55 (95% CI: −2.19 to −0.90, Z = 4.70, *P* < 0.0001, *I²* = 94%); long-term intervention (>8 weeks, 8 studies) yielded a pooled SMD of −0.37 (95% CI: −0.54 to −0.21, Z = 4.44, *P* < 0.0001, *I²* = 44%). The results of the between-group difference test (χ² = 14.28, df = 2, *P* = 0.0008, *I²* = 86.0%) demonstrated that the effects of different intervention durations were significantly distinct, and intervention duration was a crucial source of overall heterogeneity.

Further analysis revealed that medium-term intervention (5–8 weeks) had the largest absolute effect size (−1.55), followed by short-term intervention (≤4 weeks, −0.86), while long-term intervention (>8 weeks) showed a relatively weaker effect (−0.37). This suggests that medium-term intervention of 5–8 weeks may be the optimal duration for improving subthreshold depressive symptoms, and excessively long intervention duration does not lead to stronger antidepressant effects, which may be related to reduced adherence and fatigue effects associated with prolonged intervention.

##### 3.3.2.4 Subgroup analysis by age group

**Figure 6:**
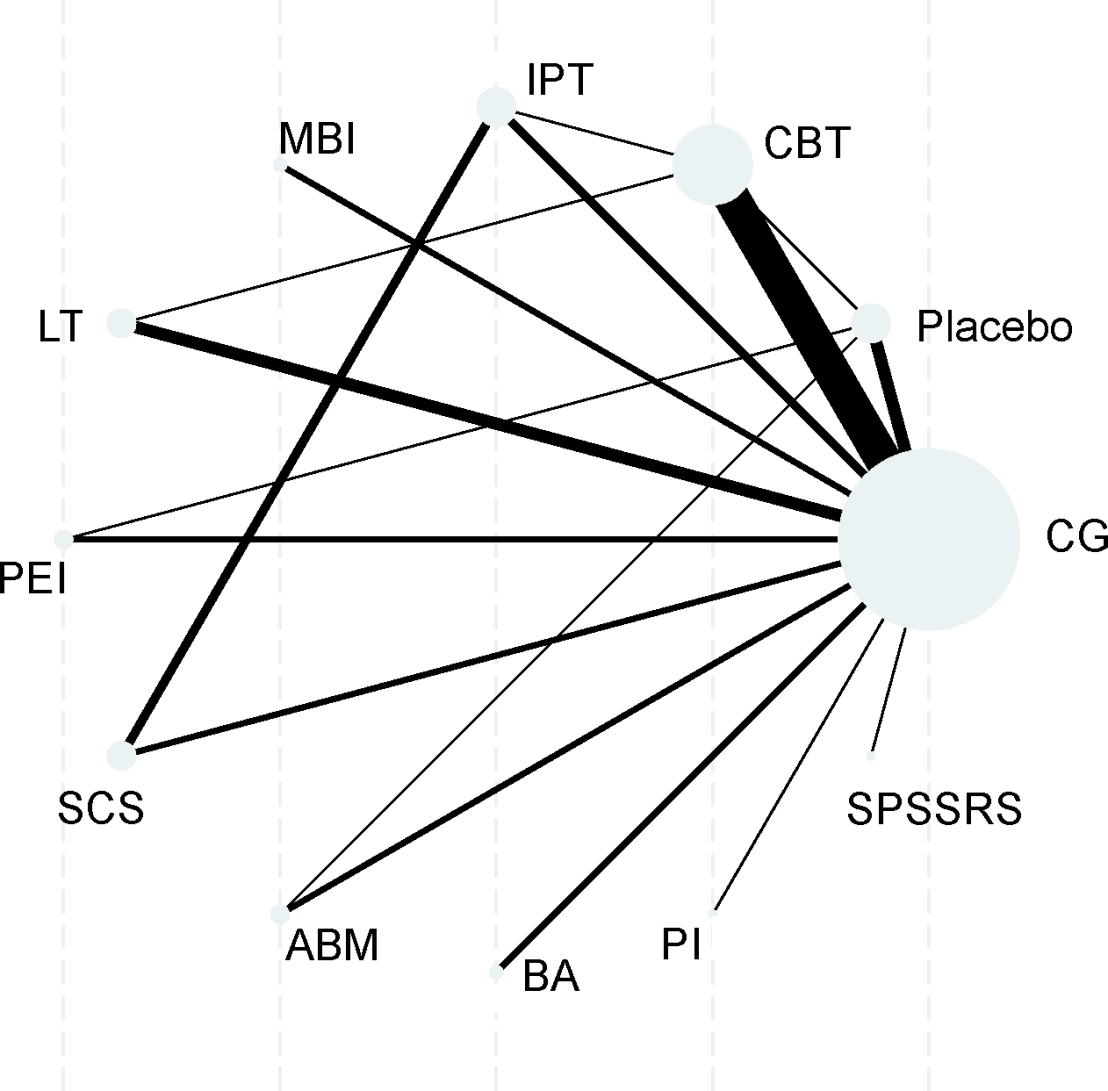
Forest plot of subgroup analysis stratified by age group.

Subgroup analyses were conducted using a random-effects model (**Figure 6**). The adolescent subgroup (11–19 years, 17 studies) yielded a pooled SMD of −0.58 (95% CI: −0.86 to −0.30, Z = 4.11, *P* < 0.0001, *I²* = 90%); the early young adult subgroup (20–25 years, 10 studies) showed a pooled SMD of −1.56 (95% CI: −2.04 to −1.08, Z = 6.32, *P* < 0.0001, *I²* = 89%). The results of the between-group difference test (χ² = 11.90, df = 1, *P* = 0.0006, *I²* = 91.6%) demonstrated that the effects of non-pharmacological interventions differed significantly between age groups, indicating that age group was a crucial source of overall heterogeneity.

Further analysis revealed that the early young adult subgroup (20–25 years) had a larger absolute effect size (−1.56) than the adolescent subgroup (11–19 years, −0.58), suggesting that non-pharmacological interventions may be more effective in improving subthreshold depressive symptoms among young adults aged 20–25 years. This difference may be related to higher intervention adherence and more mature cognitive regulation abilities in the early young adult population.

##### 3.3.2.5 Subgroup analysis by measurement tool

**Figure 7:**
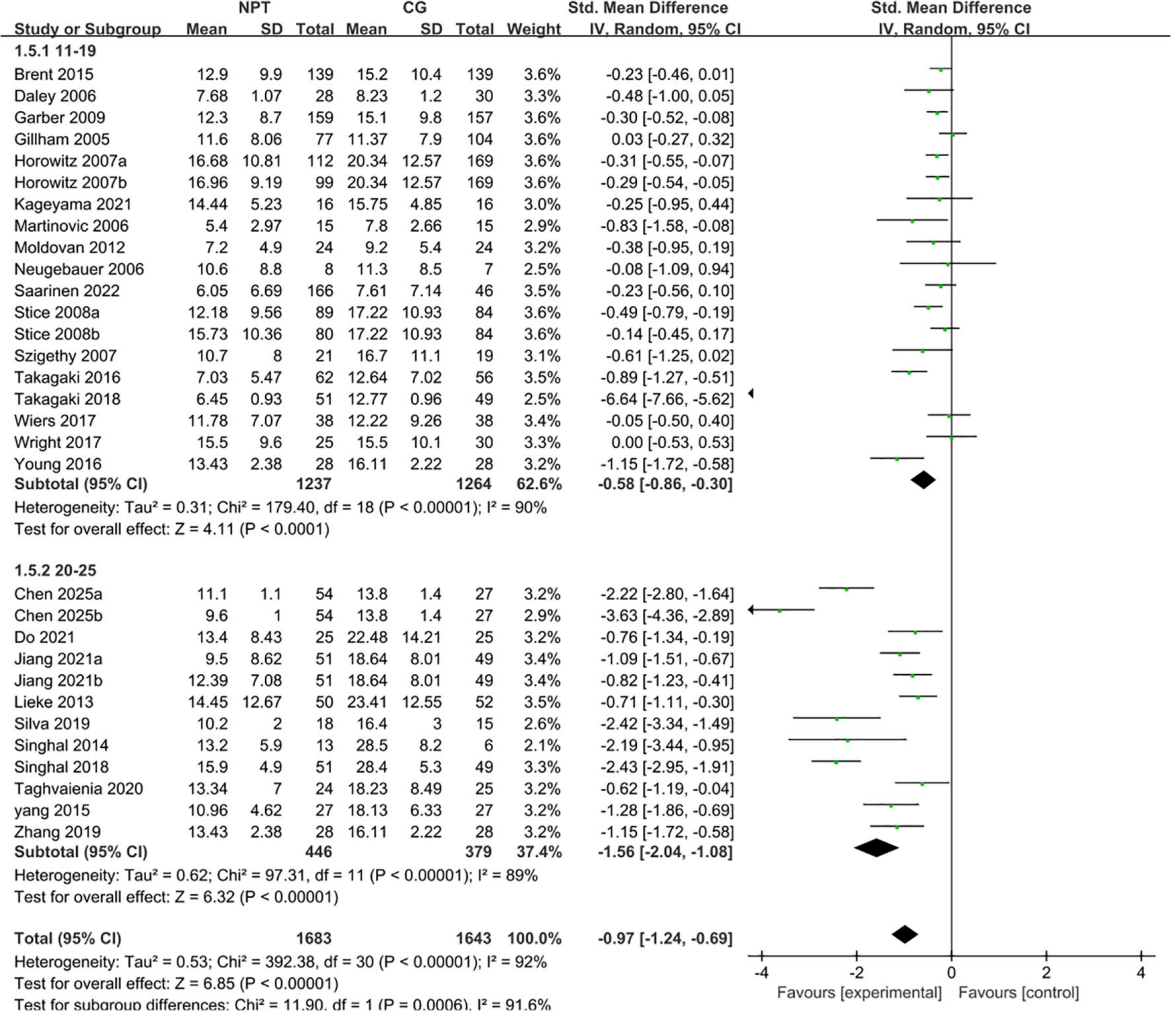
Forest plot of subgroup analysis stratified by assessment scale.

Subgroup analyses were performed using a random-effects model to assess the influence of different depressive symptom measurement tools on pooled effect sizes (**Figure 7**). A total of 27 included studies were stratified into subgroups based on the depressive symptom assessment scales extracted for the traditional meta-analysis: the BDI-II subgroup, the CES-D subgroup, the CDI subgroup, and the HAMD subgroup.

The results showed that the pooled SMD was −1.13 (95% CI: −1.58 to −0.69, Z = 4.98, *P* < 0.0001, *I²* = 95%) for the BDI-II subgroup (13 studies), −0.45 (95% CI: −0.69 to −0.21, Z = 3.68, *P* = 0.0002, *I²* = 74%) for the CES-D subgroup (7 studies), −0.60 (95% CI: −0.96 to −0.25, Z = 3.33, *P* = 0.0009, *I²* = 57%) for the CDI subgroup (6 studies), and 0.00 (95% CI: −0.53 to 0.53, *P* = 1.00) for the HAMD subgroup (1 study). The between-group heterogeneity test (χ² = 11.38, df = 3, *P* = 0.010, *I²* = 73.6%) indicated significant differences in effect sizes across measurement tool subgroups, suggesting that the type of depressive symptom assessment scale was a crucial source of overall heterogeneity in this study.

### 3.4 Network meta-analysis

A network meta-analysis of 30 randomized controlled trials was performed to compare the relative efficacy of 10 non-pharmacological interventions for adolescents with subthreshold depression. The primary outcome was depressive symptom improvement, measured by standardized scales (BDI-II, CES-D, CDI, HAMD).

#### 3.4.1 Network construction and consistency assessment

Interventions included: cognitive behavioral therapy (CBT, including iCBT, CB, family CBT), interpersonal psychotherapy (IPT, including IPTA), mindfulness/acceptance and commitment intervention (MBI, including MBT, ACT, MBSR), light therapy (LT, including BLT/DLT), physical exercise intervention (PEI), school-based social skills intervention (SCS, including SC), attention bias modification (ABM), behavioral activation (BA), positive intervention (PI), and psychosocial skills and stress reduction (SPSSRS, smartphone-based positive word stimulation).

**Figure 8:**
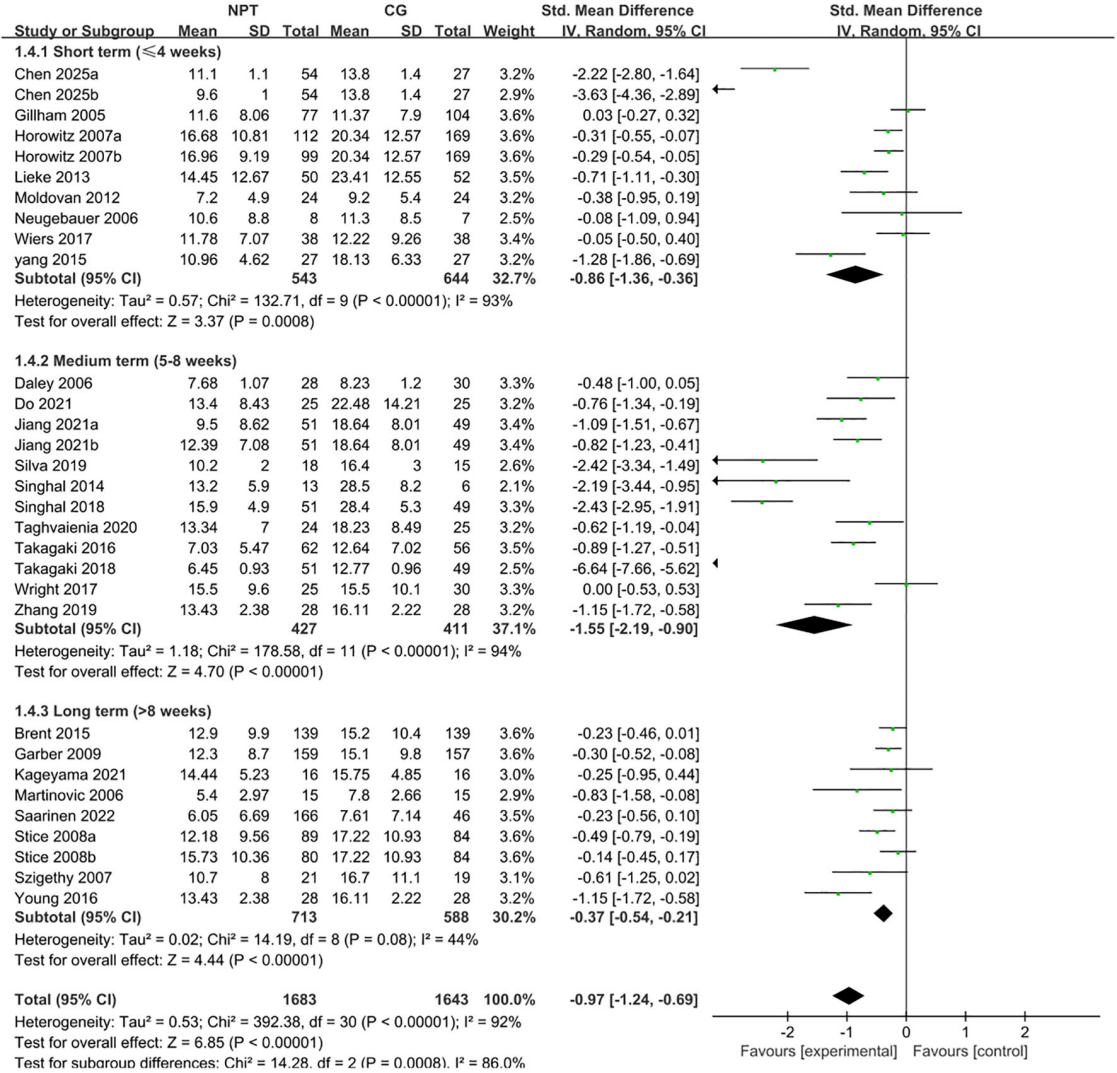
Network plot of mixed treatment comparisons for 10 non-pharmacological interventions in network meta-analysis.

A mixed treatment comparison network was constructed with usual care/waitlist control (CG, Node 1) as the reference (**Figure 8**). Node size indicated the number of studies per intervention, and edge thickness denoted direct comparison frequency. CG connected to all active interventions, with CBT, IPT, and LT forming dense connections, ensuring a complete network structure.

Consistency was assessed via the node-splitting method, with forest plots for effect visualization provided in **Appendix 3**. The global inconsistency test yielded χ²=6.72 (df=9, *P* = 0.651), confirming no significant overall inconsistency (*P*>0.05). Marginal inconsistency in a few comparison pairs did not affect conclusions, so a consistency model was used for subsequent analyses.

#### 3.4.2 Overall relative efficacy of interventions

Pooled standardized mean differences (SMDs) and corresponding 95% confidence intervals (95% CIs) were estimated for each intervention against usual care/waitlist control (CG) using the consistency model (Appendix 4). Consistent with prior findings, all active interventions demonstrated statistically significant superiority to CG, as evidenced by 95% CIs that excluded zero for all comparisons (*P* < 0.05).

Of these, BA yielded the most pronounced treatment effect, with a pooled standardized mean difference (SMD) of −3.45 (95% CI: −4.89 to −2.02) relative to the control group (CG), followed by PEI (SMD = −1.34, 95% CI: −2.68 to −0.03) and LT (SMD = −1.24, 95% CI: −2.17 to −0.31), all of which represented large effect sizes. The remaining interventions, ordered by decreasing absolute effect size (**Appendix 5**), were SCS (SMD = −1.03, 95% CI: −2.10 to 0.04), IPT (SMD = −0.92, 95% CI: −1.85 to 0.01), MBI (SMD = −0.68, 95% CI: −2.04 to 0.69), PI (SMD = −0.62, 95% CI: −2.58 to 1.35), ABM (SMD = −0.52, 95% CI: −1.83 to 0.78), CBT (SMD = −0.46, 95% CI: −1.00 to 0.08), and SPSSRS (SMD = −0.25, 95% CI: −2.26 to 1.75). Most of these interventions demonstrated moderate to moderate-to-large effect sizes, though several showed wide confidence intervals, indicative of considerable statistical uncertainty.

**Figure 9:**
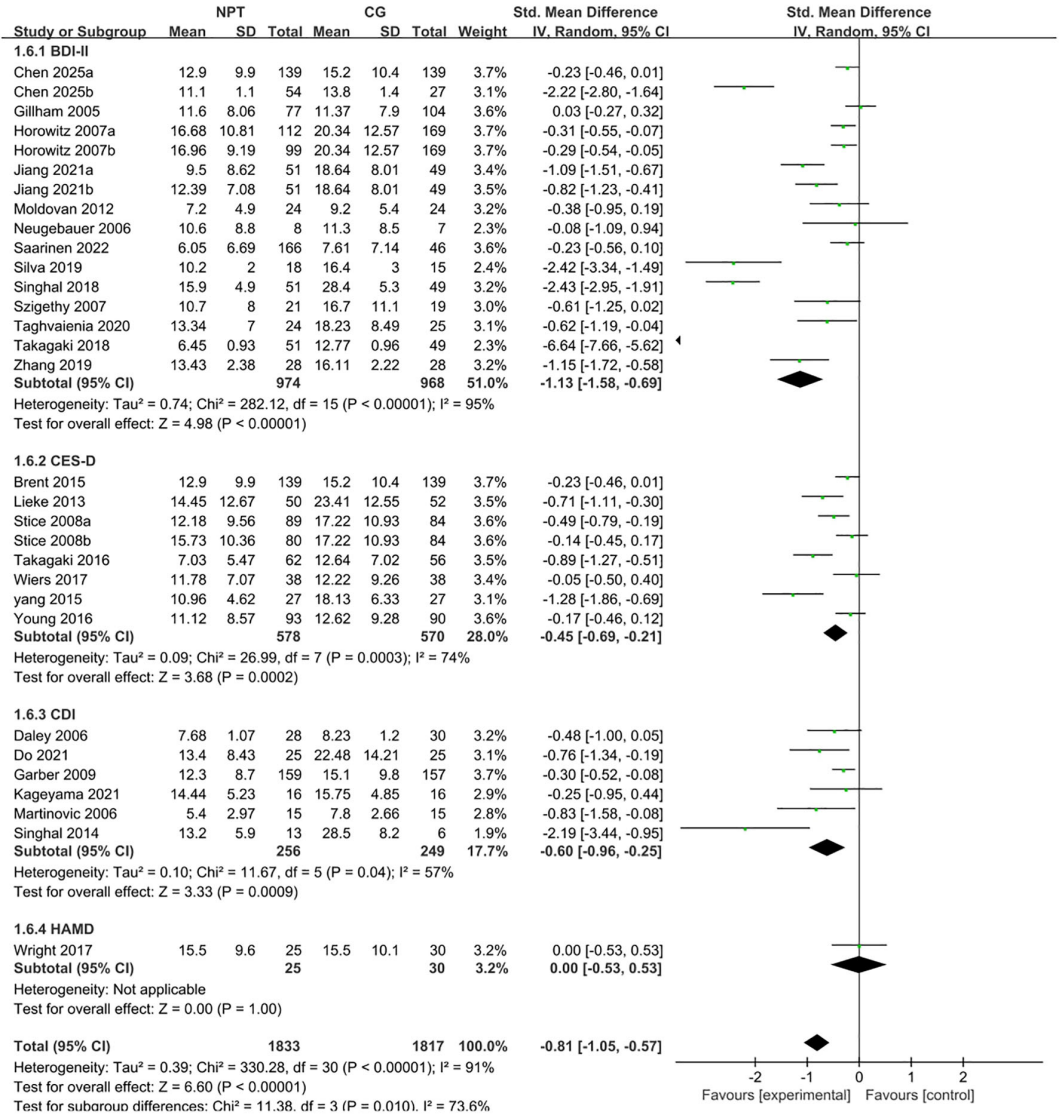
Surface under the cumulative ranking curve (SUCRA) of efficacy for non-pharmacological interventions.

**Figure 10:**
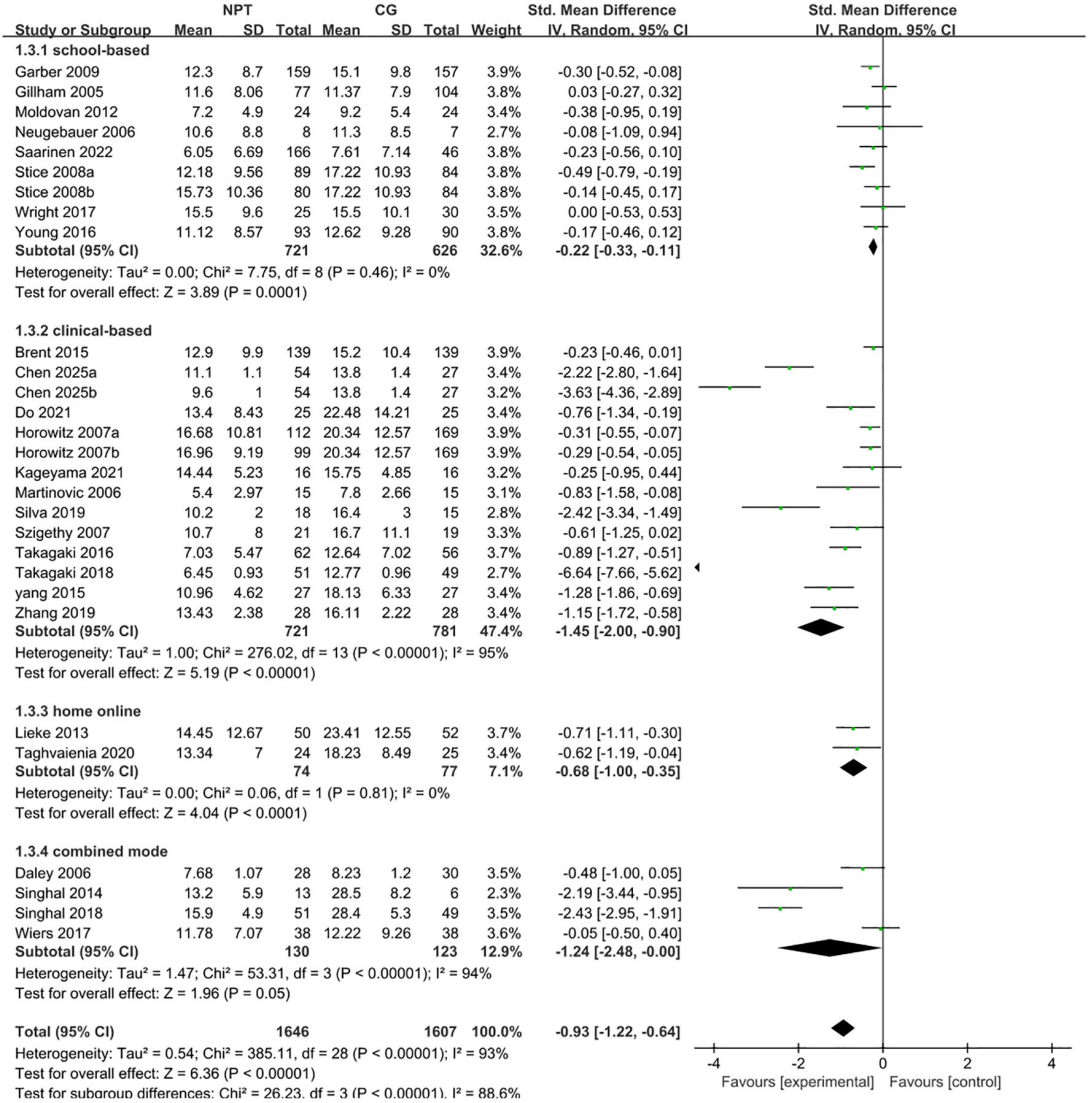
League table for pairwise comparisons of non-pharmacological interventions efficacy.

To contextualize these results, cumulative ranking probability curves were generated to visualize the probability of each intervention achieving the highest rank (**Figure 9**), alongside a league table of intervention comparisons (**Figure 10**). The BA curve occupied the upper-left region of **Figure 9**, indicating its substantially higher probability of being the most effective intervention. The LT and PEI curves overlapped closely, suggesting comparable efficacy between these two interventions. In line with effect size estimates, CG and placebo were positioned toward the right, reflecting their inferiority relative to active interventions. The league table further corroborated these patterns, with BA demonstrating consistent superiority across most comparisons and LT, SCS, and PEI comprising the next tier of effective interventions.

#### 3.4.3 Risk of bias and quality of evidence grading for included studies

**Figure 11:**
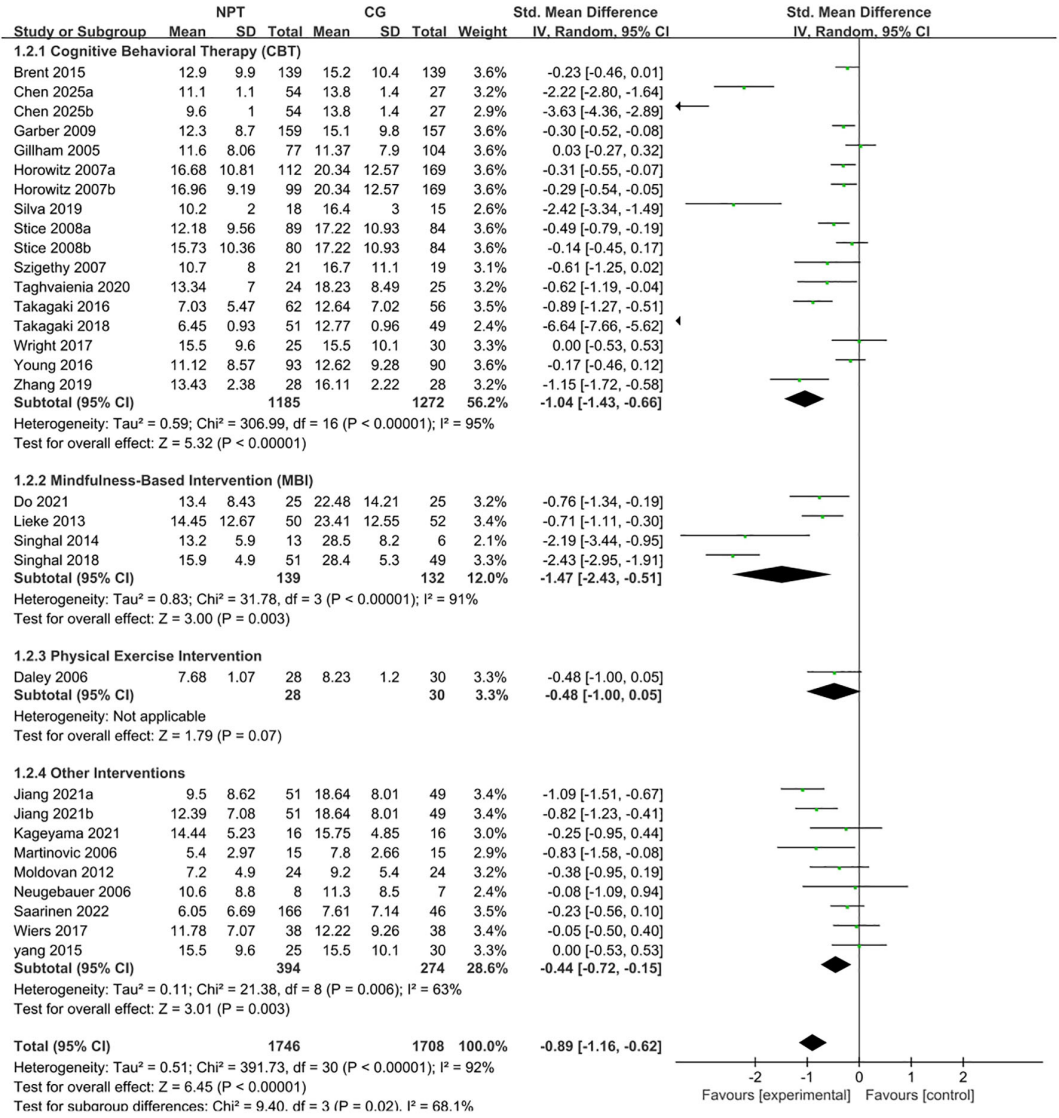
Risk of bias assessment of included studies using Cochrane RoB 2.0.

Of the 30 included randomized controlled trials, all demonstrated low risk of bias for random sequence generation. Adequate allocation concealment was reported in 12 studies, while the remaining 18 were judged to have unclear risk. For blinding of participants and personnel, 3 studies were rated as low risk, 16 as unclear, and 11 as high risk. Regarding blinding of outcome assessment, 19 studies were low risk, 2 were unclear, and 9 were high risk. All studies had low risk of incomplete outcome data. Selective reporting bias was low in 22 studies and high in 8. 29 studies were low risk for other potential biases, with 1 study rated as unclear (**Figure 11**).

Using the Confidence in Network Meta-Analysis (CINeMA) tool, the certainty of evidence for the primary outcome ranged from high to very low, with detailed results presented in **Supplementary Appendix 6.**

#### 3.4.4 Publication bias analysis

**Figure 12:**
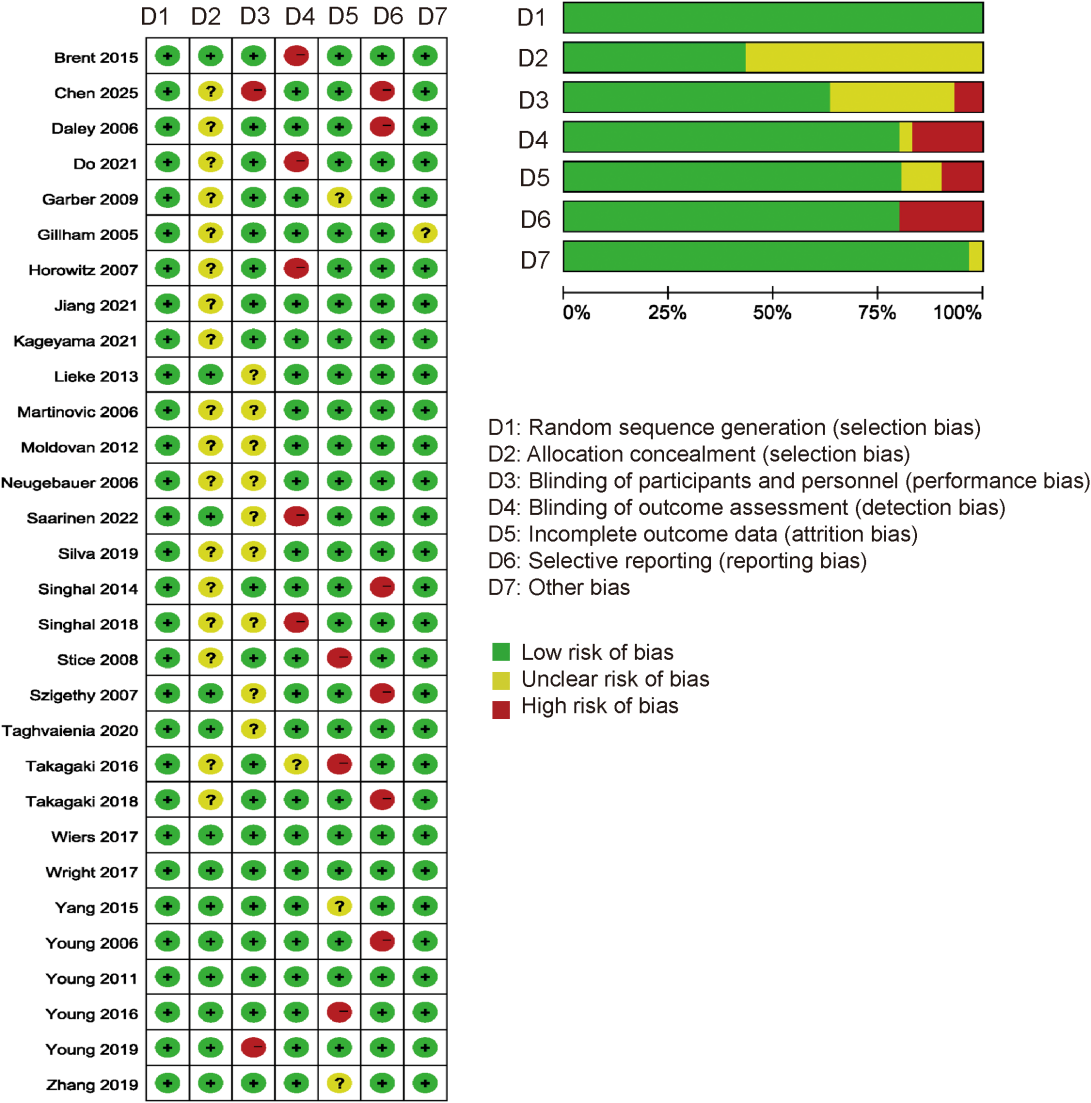
Funnel plot for publication bias assessment.

Funnel plots were generated to evaluate publication bias for the depressive symptom outcome, for which more than 10 studies were included in the present analysis (**Figure 12**). Visual inspection revealed that the effect size points were approximately symmetrically distributed around the pooled effect size, with no evident unilateral absence or asymmetric distribution observed. These findings indicated no significant publication bias among the included studies, confirming the stability of the core pooled results and the good reliability of the study conclusions.

## 4 Discussion

SD is a highly prevalent psychiatric condition among adolescents. While it does not meet the diagnostic criteria for major depressive disorder (MDD), it significantly impairs subjective well-being and psychosocial functioning, and acts as a key precursor to subsequent MDD development (52, 53). Non-pharmacological therapies are the mainstay of intervention for adolescent SD, yet the comparative efficacy of different approaches remains poorly characterized, leaving a gap in evidence-based clinical decision-making (54).Here, we conducted a systematic review and network meta-analysis (NMA) of 30 RCTs to comprehensively compare the efficacy of 10 non-pharmacological interventions for adolescent SD, and provide actionable evidence for clinical practice.

Our results demonstrated that nearly all non-pharmacological interventions were superior to control conditions (usual care/waitlist control) in alleviating depressive symptoms in adolescents with SD, consistent with prior findings (55–57). Traditional meta-analysis yielded a pooled standardized mean difference (SMD) of −0.93 (95%CI: −1.20 to −0.66), indicating a moderate-to-large effect of non-pharmacological therapies for adolescent SD and reinforcing their core role in SD management. For relative efficacy, BA exhibited the strongest overall effect (SMD = −3.45, 95%CI: −4.89 to −2.02; large effect size), followed by PEI (SMD = −1.34, 95%CI: −2.68 to −0.03) an LT (including BLT and DLT; SMD = −1.24, 95%CI: −2.17 to −0.31), all of which showed a significant therapeutic advantage with large effect sizes. The remaining interventions were ranked by efficacy from high to low as: SCS (SMD = −1.03, 95%CI: −2.10 to 0.04), IPT (SMD = −0.92, 95%CI: −1.85 to 0.01), MBI (SMD = −0.68, 95%CI: −2.04 to 0.69), PI (SMD = −0.62, 95%CI: −2.58 to 1.35), ABM (SMD = −0.52, 95%CI: −1.83 to 0.78), CBT (SMD = −0.46, 95%CI: −1.00 to 0.08), and SPSSRS (SMD = −0.25, 95%CI: −2.26 to 1.75), with most exhibiting moderate to moderate-to-large effect sizes and some showing wide confidence intervals indicative of statistical uncertainty.

This ranking differs from that of prior NMAs in adult SD populations (58)], where CBT was a top-performing intervention (SMD ≈ -0.80), in contrast to its lower ranking in our adolescent cohort. This discrepancy is likely driven by age-related developmental differences: adults benefit more from CBT’s cognitive restructuring components due to more mature cognitive functioning, while adolescents exhibit high behavioral plasticity, making BA’s behavior-focused approach and LT’s physiological regulatory mechanisms more aligned with their developmental needs. (59);Additionally, adolescent SD is more strongly linked to psychosocial stressors and circadian rhythm dysregulation—key targets of BA and LT—whereas adult SD often involves more complex etiologies (e.g., comorbid chronic illness, occupational stress), which may explain LT’s weaker performance in adult studies (51). These findings highlight the critical need to avoid generalizing adult SD intervention data to adolescents.

BA’s top ranking is attributable to its core mechanism of targeting the depression-behavioral withdrawal cycle through guided engagement in pleasant, meaningful activities to reduce avoidance and alleviate depressive symptoms (36, 41). Compared with CBT, BA requires no complex cognitive skill training, features a streamlined intervention process, and achieves higher adherence in adolescents. It is also highly adaptable to diverse settings (school, family, community), making it ideal for resource-limited primary care and school mental health services (60). In clinical practice, BA is recommended as a first-line intervention for adolescent SD, with an optimal medium-term cycle of 5–8 weeks (supported by our subgroup analysis). Core BA modules should include activity monitoring, pleasure/achievement planning, and barrier overcoming to foster sustained positive behavioral patterns.

LT and PEI ranked jointly second in relative efficacy, with SMDs of −1.24 and −1.34 respectively, both demonstrating large effect sizes relative to the control group; notably, the evidence base for both interventions was derived from a single RCT each, a critical limitation warranting cautious interpretation of their efficacy. Despite this, LT’s therapeutic potential is supported by well-characterized physiological mechanisms: it modulates hypothalamic-pituitary-adrenal axis activity and reduces neuroinflammation; elevates serotonin and catecholamine levels (61, 62); enhances mitochondrial cytochrome C oxidase (COX) activity to boost neuronal ATP synthesis and exert neuroprotective effects (63); and regulates circadian rhythms to improve sleep— a common comorbidity in adolescent SD (64). LT is easy to administer, has a short intervention cycle, and is well-tolerated, making it a suitable option for adolescents with SD and circadian sleep disturbances or seasonal mood fluctuations. Similarly, PEI exerts antidepressant effects through multiple pathways and is low-cost, non-invasive, and easily integrated into school or daily life, ideal for adolescents with poor therapy compliance or limited access to mental health services (65). Given the single-RCT evidence for both LT and PEI, clinical use requires careful individualization, and large-scale RCTs are urgently needed to validate their efficacy for adolescent SD populations.

As the most widely studied intervention for SD, CBT’s lower ranking in our analysis may stem from three factors: first, SD’s mild baseline depressive symptoms limit the utility of CBT’s cognitive restructuring core, which is more effective for moderate-to-severe depression; second, inclusion of diverse CBT subtypes (e.g., internet-based CBT, group CBT) with variable implementation fidelity may have diluted the overall effect; third, BA and LT exhibited unique advantages that were more pronounced in our multi-intervention comparison. Notably, CBT still demonstrated a stable moderate effect size and offers highly transferable cognitive skills, making it a valuable targeted intervention for adolescents with SD and comorbid anxiety or prominent cognitive biases, or as an adjunct to BA/LT to enhance long-term efficacy.

Heterogeneity testing revealed high heterogeneity across included studies (I² = 92%), driven by intervention type, duration, age subgroup, and clinical setting variability (mixed school-based prevention programs, clinical treatment trials, and inpatient interventions). Additionally, some interventions (e.g., IPT) showed marked geographical concentration (single U.S. research team, uniform protocols), potentially biasing efficacy estimation. Strict inclusion criteria ensured the NMA transitivity assumption was largely met: all participants were 11–25 years old with SD (consistent diagnostic criteria, homogeneous baselines); intervention core procedures were uniform across settings; and controls were predominantly usual care/waitlist, with no anomalous effect size deviations. Limited direct comparison data for some low-prevalence interventions, however, may compromise transitivity stability, and future studies should prioritize head-to-head comparisons to strengthen the NMA network.

This study has several important limitations. First, the evidence base for some interventions (PI, SPSRS) is weak, and LT and PEI rely on a single RCT each, resulting in limited statistical power. Second, high clinical heterogeneity exists due to mixed study settings, geographical concentration of certain interventions, a broad age range (11–25 years), and the combination of prevention- and treatment-oriented trials, which may confound efficacy estimation. Third, the transitivity assumption is potentially limited by insufficient direct comparison data for some interventions. Fourth, no long-term follow-up data were available, precluding assessment of sustained intervention effects. Fifth, evidence quality is uneven, as some studies lacked detailed reporting of random sequence generation and allocation concealment.

## 5 Conclusion

In conclusion, this NMA confirms that non-pharmacological interventions are effective for adolescents with SD, with behavioral activation (BA) demonstrating the strongest efficacy and being recommended as a first-line clinical option. The selection of clinical interventions should be individualized based on patient characteristics and account for clinical heterogeneity to optimize intervention efficacy and patient adherence. These findings provide critical evidence-based support for the standardized management of adolescent SD and offer actionable guidance for the integration of school, clinical, and community mental health services.

## Data Availability

The original contributions presented in the study are included in the article/**Supplementary Material**, further inquiries can be directed to the corresponding author/s.
