# Supplementary material for: Comparative efficacy of non-pharmacological therapies in adolescents with subthreshold depression: a systematic review and network meta-analysis
Source: Front Psychiatry. 2026 May 12;17:1799128. doi: 10.3389/fpsyt.2026.1799128 (PMC13202787; doi:10.3389/fpsyt.2026.1799128)
Supplement: Supplementary file 1 [file DataSheet1.zip › Data Sheet/Appendix 2.docx]

**Search strategies of electronic databases**

| **Database** | **Search strategy** |
| --- | --- |
| **Pubmed** | #1: "subthreshold depression"[Title/Abstract] OR "minor depression"[Title/Abstract] OR "masked depression"[Title/Abstract] OR (("smiled"[All Fields] OR "smiles"[All Fields] OR "smiling"[MeSH Terms] OR "smiling"[All Fields] OR "smile"[All Fields]) AND "Depression"[Title/Abstract]) OR "borderline depression"[Title/Abstract] OR "depression equivalent"[Title/Abstract] OR "hidden depression"[Title/Abstract] OR (("subsyndromal"[All Fields] OR "subsyndromally"[All Fields] OR "subsyndrome"[All Fields] OR "subsyndromes"[All Fields] OR "subsyndromic"[All Fields]) AND "depressive symptom"[Title/Abstract]) OR "minor depression disorder"[Title/Abstract] OR "recurrent brief depression"[Title/Abstract] OR ("Subthreshold"[All Fields] AND "depressive symptom"[Title/Abstract]) OR (("subclinic"[All Fields] OR "subclinical"[All Fields] OR "subclinically"[All Fields] OR "subclinicals"[All Fields]) AND "depressive condition"[Title/Abstract]) OR "subsyndromal depression"[Title/Abstract] OR "subclinical depression"[Title/Abstract] OR "mild depression"[Title/Abstract] OR "limited depression"[Title/Abstract] OR "subsyndromic depression"[Title/Abstract] OR "mild depression"[Title/Abstract] OR "depressive mood"[Title/Abstract] OR "minor depressive disorder"[Title/Abstract] OR "non major depression"[Title/Abstract]  #2: "adolescent"[MeSH Terms] OR "adolescent"[All Fields] OR "youth"[All Fields] OR "youths"[All Fields] OR "youth s"[All Fields] OR "adolesc"[All Fields] OR "pubert"[All Fields] OR ("women"[MeSH Terms] OR "women"[All Fields] OR "girl"[All Fields]) OR ("men"[MeSH Terms] OR "men"[All Fields] OR "boy"[All Fields]) OR ("educational status"[MeSH Terms] OR ("educational"[All Fields] AND "status"[All Fields]) OR "educational status"[All Fields] OR "schooling"[All Fields] OR "education"[MeSH Terms] OR "education"[All Fields] OR "school s"[All Fields] OR "schooled"[All Fields] OR "schools"[MeSH Terms] OR "schools"[All Fields] OR "school"[All Fields]) OR ("adolescent"[MeSH Terms] OR "adolescent"[All Fields] OR "teen"[All Fields]) OR ("underage"[All Fields] OR "underaged"[All Fields])  #3: #1 AND #2  #4 "randomized controlled trial"[Publication Type] OR "Randomized"[Title/Abstract] OR "placebo"[Title/Abstract]  #5 #3AND #4 |
| **Embase** | #1: 'subthreshold depression':ab,ti OR 'minor depression':ab,ti OR 'masked depression':ab,ti OR 'smiling depression':ab,ti OR 'borderline depression':ab,ti OR 'depression equivalent':ab,ti OR 'hidden depression':ab,ti OR 'subsyndromal depressive symptom':ab,ti OR 'minor depression disorder':ab,ti OR 'recurrent brief depression':ab,ti OR 'subthreshold depressive symptom':ab,ti OR 'subclinical depressive condition':ab,ti OR 'subsyndromal depression':ab,ti OR 'subclinical depression':ab,ti OR 'limited depression':ab,ti OR 'subsyndromic depression':ab,ti OR 'mild depression':ab,ti OR 'depressive mood':ab,ti OR 'minor depressive disorder':ab,ti OR 'non-major depression':ab,ti  #2 (((((((adolesc OR puberty OR girl OR boy OR school OR underage OR adolescents OR adolescence OR teens OR teen OR teenagers OR teenager OR youth OR youths OR adolescents,) AND female OR adolescent,) AND female OR female) AND adolescent OR female OR adolescents OR adolescents,) AND male OR adolescent,) AND male OR male) AND adolescent OR male) AND adolescents  #3 'randomized controlled trial':ti,ab,kw OR randomized:ti,ab,kw OR placebo:ti,ab,kw OR rct:ti,ab,kw  #4 #1 AND #2 AND #3 |
| **Web of science** | #1 TS=(Subthreshold depression OR minor depression OR masked depression OR smiling depression OR borderline depression OR depression equivalent OR hidden depression OR Subsyndromal Depressive Symptom OR Minor Depression Disorder OR recurrent Brief Depression OR subthreshold depressive symptom OR subclinical depressive condition OR subsyndromal depression OR subclinical depression OR mild depression OR limited depression OR subsyndromic depression OR mild depression OR depressive mood OR minor depressive disorder OR non-major depression)  #2 TS=(Youth OR adolesc OR puberty OR girl OR boy OR school OR underage OR Adolescents OR Adolescence OR Teens OR Teen OR Teenagers OR Teenager OR Youth OR Youths OR Adolescents, Female OR Adolescent, Female OR Female Adolescent OR Female OR Adolescents OR Adolescents, Male OR Adolescent, Male OR Male Adolescent OR Male Adolescents)  #3 TS=(Randomized controlled trial OR Randomized OR Placebo OR RCT)  #4 #1 AND #2 AND #3 |
| **Cochrane** | #1 (subthreshold OR 'sub threshold' OR subclinical OR 'sub clinical' OR subsyndromal OR 'sub syndromal' OR brief) AND recurrent OR 'minors'/exp OR minors OR 'minor'/exp OR minor  #2 ((depressi* OR depression OR depressive) AND disorder OR mood) AND disorders  #3: #1 AND #2  #4 Youth OR adolesc OR puberty OR girl OR boy OR school OR underage OR Adolescents OR Adolescence OR Teens OR Teen OR Teenagers OR Teenager OR Youth OR Youths OR Adolescents, Female OR Adolescent, Female OR Female Adolescent OR Female OR Adolescents OR Adolescents, Male OR Adolescent, Male OR Male Adolescent OR Male Adolescents  #5 3 AND #4  #6 Randomized controlled trial OR Randomized OR Placebo OR RCT  #7 #5 AND #6 |
| **PsycINFO** | #1 Subthreshold depression OR minor depression OR masked depression OR smiling depression OR borderline depression OR depression equivalent OR hidden depression OR Subsyndromal Depressive Symptom OR Minor Depression Disorder OR recurrent Brief Depression OR subthreshold depressive symptom OR subclinical depressive condition OR subsyndromal depression OR subclinical depression OR mild depression OR limited depression OR subsyndromic depression OR mild depression OR depressive mood OR minor depressive disorder OR non-major depression  #2 Youth OR adolesc OR puberty OR girl OR boy OR school OR underage OR Adolescents OR Adolescence OR Teens OR Teen OR Teenagers OR Teenager OR Youth OR Youths OR Adolescents, Female OR Adolescent, Female OR Female Adolescent OR Female OR Adolescents OR Adolescents, Male OR Adolescent, Male OR Male Adolescent OR Male Adolescents  #3 Randomized controlled trial OR Randomized OR Placebo OR RCT  #4 #1 AND #2 AND #3 |
| **CINAHL** | #1 Subthreshold depression OR minor depression OR masked depression OR smiling depression OR borderline depression OR depression equivalent OR hidden depression OR Subsyndromal Depressive Symptom OR Minor Depression Disorder OR recurrent Brief Depression OR subthreshold depressive symptom OR subclinical depressive condition OR subsyndromal depression OR subclinical depression OR mild depression OR limited depression OR subsyndromic depression OR mild depression OR depressive mood OR minor depressive disorder OR non-major depression  #2 Youth OR adolesc OR puberty OR girl OR boy OR school OR underage OR Adolescents OR Adolescence OR Teens OR Teen OR Teenagers OR Teenager OR Youth OR Youths OR Adolescents, Female OR Adolescent, Female OR Female Adolescent OR Female OR Adolescents OR Adolescents, Male OR Adolescent, Male OR Male Adolescent OR Male Adolescents  #3 Randomized controlled trial OR Randomized OR Placebo OR RCT  #4 #1 AND #2 AND #3 |
