# Supplementary material for: Comparative efficacy of non-pharmacological therapies in adolescents with subthreshold depression: a systematic review and network meta-analysis
Source: Front Psychiatry. 2026 May 12;17:1799128. doi: 10.3389/fpsyt.2026.1799128 (PMC13202787; doi:10.3389/fpsyt.2026.1799128)
Supplement: Supplementary file 1 [file DataSheet1.zip › Data Sheet/Appendix 3.docx]

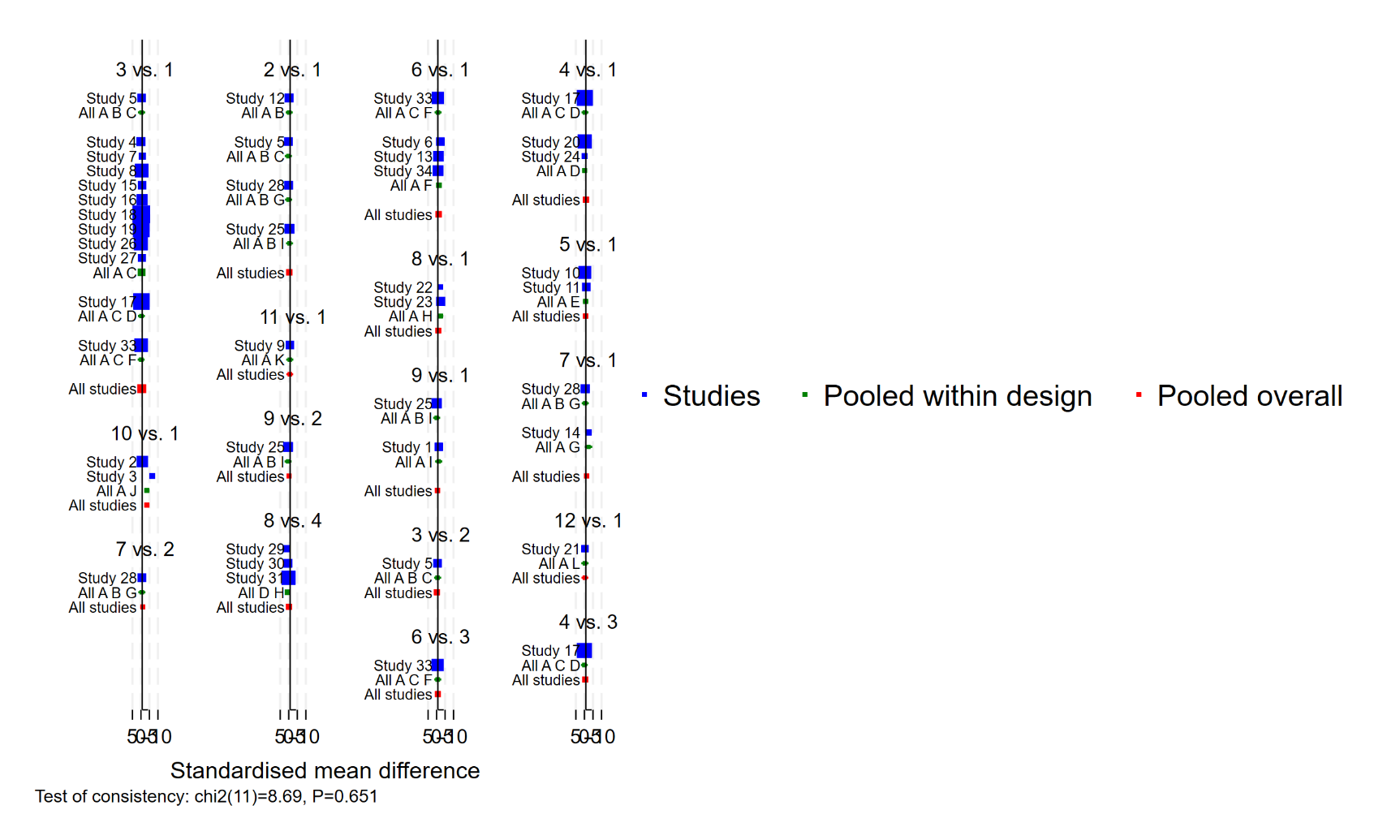


Note: Corresponding codes and abbreviations for interventions: 1 Control group (CG); 2 Placebo; 3 Cognitive behavioral therapy (CBT); 4 Interpersonal psychotherapy (IPT); 5 Mindfulness/acceptance and commitment intervention (MAC); 6 Light therapy (LT); 7 Physical exercise intervention (PEI); 8 Self-compassion intervention; 9 Attentional bias modification (ABM); 10 Behavioral activation (BA); 11 Positive intervention (PI); 12 Psychosocial skills and stress reduction (SPSSRS).
