# Supplementary material for: Comparative efficacy of non-pharmacological therapies in adolescents with subthreshold depression: a systematic review and network meta-analysis
Source: Front Psychiatry. 2026 May 12;17:1799128. doi: 10.3389/fpsyt.2026.1799128 (PMC13202787; doi:10.3389/fpsyt.2026.1799128)
Supplement: Supplementary file 1 [file DataSheet1.zip › Data Sheet/Appendix 4.docx]

Table. Treatment Relative Ranking Based on SUCRA Values

| Treatment | SUCRA (%) | PrBest (%) | MeanRank | Ranking |
| --- | --- | --- | --- | --- |
| BA | 99.6 | 96.4 | 1 | 1 |
| PEI | 70.8 | 1.4 | 4.2 | 2 |
| LT | 69.9 | 0.3 | 4.3 | 3 |
| SCS | 61.6 | 0.2 | 5.2 | 4 |
| IPT | 57.5 | 0.1 | 5.7 | 5 |
| MBI | 46.5 | 0.2 | 6.9 | 6 |
| PI | 42.8 | 0.9 | 7.3 | 7 |
| ABM | 39.9 | 0.2 | 7.6 | 8 |
| CBT | 36.8 | 0 | 8 | 9 |
| SPSRS | 33 | 0.2 | 8.4 | 10 |
| Placebo | 28.4 | 0 | 8.9 | 11 |
| CG | 13.4 | 0 | 10.5 | 12 |
