# Supplementary material for: Comparative efficacy of non-pharmacological therapies in adolescents with subthreshold depression: a systematic review and network meta-analysis
Source: Front Psychiatry. 2026 May 12;17:1799128. doi: 10.3389/fpsyt.2026.1799128 (PMC13202787; doi:10.3389/fpsyt.2026.1799128)
Supplement: Supplementary file 1 [file DataSheet1.zip › Data Sheet/Appendix 5.docx]

| Intervention | SMD | 95% CI (LCI, UCI) |
| --- | --- | --- |
| BA vs CG | -3.45 | (-4.89, -2.02) |
| PEI vs CG | -1.34 | (-2.68, -0.03) |
| LT vs CG | -1.24 | (-2.17, -0.31) |
| SCS vs CG | -1.03 | (-2.10, 0.04) |
| IPT vs CG | -0.92 | (-1.85, 0.01) |
| MBI vs CG | -0.68 | (-2.04, 0.69) |
| PI vs CG | -0.62 | (-2.58, 1.35) |
| ABM vs CG | -0.52 | (-1.83, 0.78) |
| CBT vs CG | -0.46 | (-1.00, 0.08) |
| SPSRS vs CG | -0.25 | (-2.26, 1.75) |
| Placebo vs CG | -0.28 | (-1.19, 0.63) |
