# Supplementary material for: Comparative efficacy of non-pharmacological therapies in adolescents with subthreshold depression: a systematic review and network meta-analysis
Source: Front Psychiatry. 2026 May 12;17:1799128. doi: 10.3389/fpsyt.2026.1799128 (PMC13202787; doi:10.3389/fpsyt.2026.1799128)
Supplement: Supplementary file 1 [file DataSheet1.zip › Data Sheet/Appendix 6. CINEMA.docx]

**Evaluation of the Certainty of evidence Using CINEMA Framework, mixed evidence**

| **Comparison** | **Number of studies** | **Within-study bias** | **Reporting bias** | **Indirectness** | **Imprecision** | **Heterogeneity** | **Incoherence** | **Confidence rating** |
| --- | --- | --- | --- | --- | --- | --- | --- | --- |
| ABM: CG | 1 | No concerns | Low risk | No concerns | No concerns | No concerns | No concerns | High |
| BA: CG | 2 | Some concerns | Low risk | No concerns | No concerns | No concerns | Some concerns | Low |
| BLT: CG | 1 | No concerns | Low risk | No concerns | No concerns | No concerns | Some concerns | Moderate |
| CB: CG | 2 | No concerns | Low risk | No concerns | Major concerns | No concerns | No concerns | Low |
| CBT: CG | 2 | No concerns | Low risk | No concerns | No concerns | Major concerns | No concerns | Low |
| CG: DLT | 1 | No concerns | Low risk | No concerns | No concerns | No concerns | Some concerns | Moderate |
| CG: IPTA | 1 | No concerns | Low risk | Some concerns | Major concerns | No concerns | Some concerns | Very low |
| CG: MBT | 2 | No concerns | Low risk | No concerns | No concerns | Major concerns | Some concerns | Low |
| CG: PI | 1 | Some concerns | Low risk | No concerns | No concerns | Major concerns | Some concerns | Very low |
| CG: Placebo | 2 | No concerns | Low risk | No concerns | Major concerns | No concerns | No concerns | Low |
| CG: iCBT | 1 | No concerns | Low risk | Some concerns | Major concerns | No concerns | Some concerns | Very low |

**Evaluation of the Certainty of evidence Using CINEMA Framework, mixed evidence**

| **Comparison** | **Number of studies** | **Within-study bias** | **Reporting bias** | **Indirectness** | **Imprecision** | **Heterogeneity** | **Incoherence** | **Confidence rating** |
| --- | --- | --- | --- | --- | --- | --- | --- | --- |
| CBT: CG | 7 | No concerns | Low risk | No concerns | No concerns | Major concerns | Major concerns | Very low |
| CG: EA | 1 | Some concerns | Low risk | Some concerns | No concerns | Major concerns | Major concerns | Very low |
| CG: GCBT | 2 | Some concerns | Low risk | No concerns | Major concerns | No concerns | Major concerns | Very low |
| CG: IPT | 1 | No concerns | Low risk | Some concerns | Major concerns | No concerns | No concerns | Low |
| CG: IPTA | 1 | No concerns | Low risk | No concerns | Major concerns | No concerns | Major concerns | Very low |
| CG: SCS | 2 | No concerns | Low risk | No concerns | No concerns | No concerns | Major concerns | Low |
| CG: SPSRS | 1 | No concerns | Low risk | No concerns | Major concerns | No concerns | Major concerns | Very low |
| CG: iCBT | 1 | No concerns | Low risk | No concerns | Major concerns | No concerns | Major concerns | Very low |
