# Supplementary material for: Comparative efficacy of non-pharmacological therapies in adolescents with subthreshold depression: a systematic review and network meta-analysis
Source: Front Psychiatry. 2026 May 12;17:1799128. doi: 10.3389/fpsyt.2026.1799128 (PMC13202787; doi:10.3389/fpsyt.2026.1799128)
Supplement: Supplementary file 1 [file DataSheet1.zip › Data Sheet/Table S2.docx]

**Table S2**

| **Study ID** | **Definition of subthreshold depression** | **Assessment tool** | **Cutoff score** | **Method to exclude MDD** | **Intervention Type** | **Delivery Mode** | **Course of treatment** | **Core Components** | **Provider Background** | **Adherence Monitoring** |
| --- | --- | --- | --- | --- | --- | --- | --- | --- | --- | --- |
| Gillham 2005 | Elevated depressive symptoms without current major depressive disorder (MDD) | CDI | Girls ≥7; Boys ≥9 | Excluded those meeting K-SADS-PL criteria for MDD/dysthymia; two-stage diagnostic interview (DICA-R + K-SADS-PL) | CBT | In-school group | 4 weeks | Problem-solving training, cognitive restructuring, social skills training, emotion regulation guidance | School counselors (MA) | Attendance records + post-session questionnaires |
| Martinovic 2006 | Subthreshold depressive disorder; mild depressive symptoms without DSM-IV MDD diagnosis | BDI, CES-D, HAMD | BDI ≥6–8; CES-D ≥9–14; HAMD 6–8 | Excluded MDD, dysthymia, bipolar disorder, psychosis via Kiddie-SADS-E-R and adult structured interview | CB | Community clinic | 6 months | Emotional validation, active listening, stress management techniques, goal-setting exercises | Clinical psychologists | Session logs + monthly follow-up calls |
| Neugebauer 2006 | Subsyndromal depression (SD); clinically significant depressive symptoms without current MDD | HAMD-17 | >7 | Excluded via SCID-IV-CV structured interview; no current MDD | IPT | After-school group | 4 weeks | Depression-related psychoeducation, sleep hygiene guidance, healthy coping strategy training, social support network building | Trained educators | Attendance tracking + parent-reported adherence |
| Young 2006 | Subthreshold depression; ≥2 depressive symptoms but not meeting MDD criteria | CES-D | ≥16 | Excluded via K-SADS-PL; no current MDD, dysthymia, bipolar disorder, or psychosis | IPTA | In-school group | 6 months | Interpersonal role-play, communication skills training, conflict resolution techniques, relationship adjustment guidance | School psychologists | Participation checklists + adherence surveys |
| Daley 2006 | Elevated depressive symptoms without formal MDD diagnosis | CDI | ≥13 | Excluded those with diagnosed major psychiatric disorders; no formal MDD diagnosis | PEI | Clinic + home-based | 8 weeks | Aerobic exercise (walking/jogging), strength training, daily activity logging, motivational coaching | Exercise physiologists | Activity trackers + weekly progress reports |
| Horowitz 2007 | Adolescents with subclinical/subthreshold depressive symptoms (indicated prevention sample) | CDI, CES-D | Elevated depressive symptoms | No current MDD; sample selected as indicated prevention for subsyndromal symptoms | CBT, IPT | University outpatient | 8 weeks | Cognitive restructuring, exposure therapy, relaxation training, relapse prevention planning (CBT); interpersonal inventory sorting, grief processing (IPT) | Licensed psychologists | Session notes + post-intervention interviews |
| Szigethy 2007 | Mild to moderate subsyndromal depression; no current MDD/dysthymia | CDI (child + parent) | ≥9 | Excluded MDD, dysthymia, bipolar disorder, psychosis via DSM-IV K-SADS-PL interview | CBT | Hospital clinic | 12 weeks | Parent-child communication training, family problem-solving exercises, adolescent cognitive restructuring | Child psychologists + family therapists | Family attendance + feedback forms |
| Stice 2008 | Elevated depressive symptoms without current MDD | CES-D | ≥20 | Excluded those with current MDD via K-SADS diagnostic interview | CBT, CB | In-school group | 6 months | Body image distortion correction, cognitive restructuring, self-esteem building, media literacy training (CBT); emotional sharing, peer support (CB) | Psychology-major research assistants | Participation logs + body image scales |
| Garber 2009 | Subsyndromal depressive symptoms or prior depression in remission; no current MDD | CES-D | ≥20 | Excluded current DSM-IV mood disorder via K-SADS-PL structured diagnostic interview | CBT | In-school/after-school | 8 weeks | Cognitive restructuring, parent-adolescent joint sessions, mood monitoring, social skills development | School psychologists | Attendance records + multi-informant reports |
| Young 2011 | Subthreshold depressive symptoms; ≥2 depressive symptoms but no current MDD/dysthymia | CES-D | ≥16 | Excluded via K-SADS-PL structured interview; no current MDD or dysthymia | -IPTA | After-school group | 6 months | Interpersonal inventory sorting, grief processing, role transition adaptation, communication skills training | Mental health workers | Checklists + reflection records |
| Moldovan 2012 | Subthreshold depression (mild depressive symptomatology) | BDI-II | 10–16 | Excluded those receiving current psychotherapy or psychotropic medication; BDI-II >16 were excluded/referred | CB, Placebo | After-school group | 1 month | Emotional sharing, peer support, positive reinforcement, stress coping techniques (CB); inactive control with no active intervention (Placebo) | School counselors | Attendance + peer-reported participation |
| Lieke 2013 | Adolescent girls with elevated subclinical/subthreshold depressive symptoms not meeting full MDD criteria | CDI, CES-D | CDI ≥16 | Excluded those receiving mental health care; CDI >19 with suicidal ideation were referred and excluded | CBT | Home-based online | 4 weeks | Guided meditation, breathing exercises, mindfulness journaling, cognitive restructuring | Licensed mindfulness instructors | Module completion rate + practice logs |
| Singhal 2014 | At-risk/subclinical depressive symptoms not meeting full MDD criteria | CDI, CES-DC | CDI ≥14; CES-DC elevated | Excluded those with formal MDD diagnosis; sample selected as indicated prevention for subsyndromal symptoms | SCS | University campus | 8 weeks | Hatha yoga practice, mindfulness meditation, stress reduction training, self-compassion exercises | Certified yoga instructors | Attendance + practice records |
| Yang 2015 | Elevated depressive symptoms without current MDD | BDI-II | ≥14 | Excluded via SCID-IV structured interview; no current major depressive episode | ABM | University lab | 4 weeks | Negative stimulus visual search tasks, attention bias correction training, real-time reaction time feedback | Research psychologists | Task completion rate + assessment tests |
| Brent 2015 | Subsyndromal depressive symptoms or remitted depression; no current major depressive episode | CES-D | ≥20 | Excluded via K-SADS-PL/SCID-I structured interview; no current MDD/dysthymia | CBT | University/community | 3 months | Cognitive restructuring, suicide risk assessment, family coordination support, emotion regulation training | Child psychiatrists + psychologists | Session logs + risk monitoring |
| Takagaki 2016 | Clinically significant depressive symptoms not meeting full MDD criteria | BDI-II | ≥10 | Excluded via CIDI structured interview; no MDD episode in the past year | BA | University health center | 5 weeks | Depression psychoeducation, activity monitoring, behavioral hierarchy construction, behavioral experiments, positive activity scheduling | Health center psychologists | Attendance + feedback forms |
| Young 2016 | Elevated depressive symptoms; ≥2 depressive symptoms (mood/irritability/anhedonia) but no current MDD/dysthymia | CES-D | ≥16 | Excluded via K-SADS-PL structured diagnostic interview; no current MDE or dysthymia | IPTA | In-school/after-school | 6 months | Interpersonal role-play, social skills training, peer support groups, cognitive restructuring | School psychologists | Participation logs + teacher-reported adherence |
| Wright 2017 | Adolescents with elevated depressive symptoms/low mood without current major depressive episode | MFQ, BDI | MFQ ≥20 | Excluded active psychosis and severe depression requiring specialist treatment; confirmed no current MDE | iCBT | In-school group | 8 weeks | Acceptance exercises, values clarification, mindfulness practice, committed action planning, online cognitive restructuring | Certified ACT therapists | Attendance + process measures |
| Wiers 2017 | Elevated depressive symptoms in adolescents without formal MDD diagnosis | CDI | >7 | No formal MDD diagnosis; only subsyndromal/symptomatic youth included | ABM, Placebo | Home-based online + school assessment | 4 weeks | Emotional stimulus visual search, attention training, real-time feedback (ABM); inactive control with no bias correction (Placebo) | Research team + school staff | Module completion rate + tests |
| Takagaki 2018 | Depressive symptoms below the threshold for full MDD diagnosis | BDI-II | ≥10 | Excluded via CIDI structured interview; no current or recent major depressive episode | BA | University health center | 5 weeks | Emotional support, problem-solving guidance, positive activity scheduling, high-stress self-management planning | Health center counselors | Session logs + follow-up |
| Singhal 2018 | Subclinical depression; elevated depressive symptoms without full MDD diagnosis | CDI, CES-DC | CDI 14–24 | Excluded via clinical assessment; no current MDD; sample defined as subclinical non-MDD | SCS | University campus | 8 weeks | Hatha yoga practice, guided meditation, group reflection, self-compassion training | Certified instructors | Attendance + practice logs |
| Silva 2019 | Mild to moderate depressive symptoms in adolescents with ADHD; no current MDD | CDI | ≥17 | Excluded via clinical assessment; no DSM-IV diagnosis of MDD | PEI | University health center | 8 weeks | Aerobic exercise training, activity logging, motivational coaching, mood monitoring | Health center psychologists | Participation checklists + surveys |
| Young 2019 | Elevated depressive symptoms; ≥2 depressive symptoms (mood/anhedonia/irritability) but no current MDD/dysthymia | CES-D | ≥16 | Excluded via K-SADS-PL diagnostic interview; no current MDD or dysthymia | IPTA | In-school/after-school | 6 months | Interpersonal communication training, family involvement sessions, cognitive restructuring, school environment adjustment guidance | School + family counselors | Attendance + multi-informant reports |
| Zhang 2019 | Subthreshold (subsyndromal/subclinical) depression; depressive symptoms not meeting DSM-IV MDD criteria | BDI-II | 14–19 | Excluded via SCID structured diagnostic interview; no current MDD, bipolar disorder, or psychotic disorders | MBT | University counseling center | 8 weeks | Guided meditation, body scan exercises, mindfulness practice, academic stress management, interpersonal skills training | Counseling center psychologists | Logs + satisfaction surveys |
| Taghvaienia 2020 | Mild-to-moderate depressive symptoms; subsyndromal/subthreshold depression | BDI-II | 14–28 | Excluded those meeting DSM-5 criteria for MDD | PI | Home-based online | 8 weeks | Positive psychology exercises, strength recognition training, mood tools, coping videos, peer support forums | Licensed psychologists (online) | Module completion rate + activity logs |
| Jiang 2021 | Non-seasonal subthreshold depression; 2–4 depressive symptoms ≥2 weeks without meeting MDD criteria | HAMD-24, BDI-II, CES-D | HAMD-24: 8–20; BDI-II ≥14; CES-D ≥16 | Excluded via psychiatrist interview; DSM-5 criteria used to rule out MDD, bipolar disorder, psychosis | BLT | University dormitories (self-administered) | 8 weeks | 10,000-lux bright light exposure, sleep-wake cycle monitoring, exposure time guidance, circadian rhythm regulation | Research team (remote guidance) | Light logs + sleep diaries |
| Do 2021 | Adolescents with clinically significant mild depressive symptoms without current MDD | PHQ-9, CES-D | PHQ-9 ≥5; CES-D ≥16 | Excluded those with diagnosed MDD; no current psychiatric disorder treatment | iCBT | University mental health center | 8 weeks | Online cognitive restructuring, meditation, body scan, walking meditation, group discussion | Certified MBSR instructors | Attendance + practice logs |
| Kageyama 2021 | Subthreshold depression (SD); clinically significant depressive symptoms without current MDD | CES-D | ≥16 | Excluded via MINI diagnostic interview; no current/past MDE; no lifetime psychiatric disorders | SPSRS | Community mental health center | 5 weeks | Subliminal + supraliminal positive word stimulation via videos, confidence-boosting vocabulary presentation, YouTube video selection with filtered content | Community mental health workers | Session logs + follow-up |
| Saarinen 2022 | Adolescents with at least mild clinically significant depressive symptoms without current MDD | RBDI (modified BDI) | ≥5 | Excluded those meeting diagnostic criteria for current MDD; no formal psychiatric disorder diagnosis | MBT | In-school group | 9 weeks | Mindfulness meditation, depression cognition education, emotional literacy training, help-seeking behavior promotion | School counselors + educators | Attendance + knowledge tests |
| Chen 2025 | Clinically significant depressive symptoms not meeting DSM-5 MDD criteria | PHQ-9 | 5–19 | Excluded those with severe depression or current MDD via clinical assessment; no current mental disorder diagnosis | LT (BLT, DLT) | University counseling center | 2 weeks | Bright light therapy (BLT), dim light therapy (DLT), light exposure scheduling, sleep-wake cycle regulation | University counseling center psychologists (master’s level or above) | Session attendance records + weekly mood logs + post-intervention adherence questionnaires |
| **Abbreviations**: CDI: Children’s Depression Inventory; CES-D: Center for Epidemiologic Studies Depression Scale; CES-DC: Center for Epidemiologic Studies Depression Scale for Children; BDI: Beck Depression Inventory; BDI-II: Beck Depression Inventory-II; HAMD: Hamilton Rating Scale for Depression; PHQ-9: Patient Health Questionnaire-9; MFQ: Mood and Feelings Questionnaire; MDD: Major Depressive Disorder; MDE: Major Depressive Episode; DSM: Diagnostic and Statistical Manual of Mental Disorders; SCID: Structured Clinical Interview for DSM Disorders; K-SADS-PL: Kiddie Schedule for Affective Disorders and Schizophrenia for School-Age Children; CIDI: Composite International Diagnostic Interview; MINI: Mini-International Neuropsychiatric Interview; Subthreshold Depression; ADHD: Attention-Deficit/Hyperactivity Disorder | | | | | | | | | | |
